# Numerical investigation of the effect of air layer on drag reduction in channel flow over a superhydrophobic surface

**DOI:** 10.1038/s41598-024-63070-3

**Published:** 2024-05-27

**Authors:** Hoai-Thanh Nguyen, Sang-Wook Lee, Jaiyoung Ryu, Minjae Kim, Jaemoon Yoon, Kyoungsik Chang

**Affiliations:** 1 School of Mechanical Engineering, University of Ulsan, Ulsan, 44610 South Korea; 2 School of Mechanical Engineering, Korea University, Seoul, 02841 South Korea; 3 Agency for Defense Development, Changwon, 51678 South Korea

**Keywords:** Superhydrophobic surface, Drag reduction, DNS, OpenFOAM, Slip velocity, Engineering, Physics

## Abstract

This study investigates the effects of an air layer on drag reduction and turbulence dynamics in channel flow over a superhydrophobic surface (SHS). Employing the OpenFOAM platform, direct numerical simulation was conducted to investigate turbulent channel flow with an air layer over an SHS. The simulations, which take into account the interaction between water and air, analyze various parameters such as velocity distribution, drag reduction (DR), Reynolds stress, turbulent kinetic energy (TKE), and coherent structures near the water–air interface. The presence of an air layer significantly alters the velocity distribution, leading to higher velocities at the interface compared to simulations without the air layer. Notably, the thickness of the air layer emerges as an important factor, with larger thicknesses resulting in increased velocities and drag reduction. This study underscores the substantial impact of the air layer on TKE near the superhydrophobic surface, emphasizing its role in understanding and optimizing drag reduction. Furthermore, the nonlinear relationship between slip velocity, Q contours, and coherent structures near the SHS are investigated.

## Introduction

Fuel economy and efficient fuel utilization are major concerns in modern industries, especially for vehicles such as ships, submarines, automobiles, and airplanes. Skin friction reduction is one of the methods employed to decrease the drag on moving objects, thereby conserving fuel. Numerous methods for skin friction reduction have previously been studied, which included grooved^1^, flexible walls^2^, microbubbles^3^, and superhydrophobic surfaces (SHS)^4^. In this study, we apply the superhydrophobic surface method to reduce skin drag. Previous studies of superhydrophobic surfaces have primarily focused on textures made of ridge or post type^5–7^. According to previous studies, superhydrophobic surface textures trap air to create an air-cushioning effect and reduce frictional resistance between the liquid and the coated surface. The widely accepted mechanism of drag reduction on superhydrophobic surfaces is the theory of slip length. This theory suggests that water causes the wall slip to slide across the superhydrophobic surface, reducing the gradient of the velocity in the boundary layer, decreasing the Reynolds shear stress, and slowing down the change of laminar adhesion surface. Studies on the drag reduction on superhydrophobic surfaces in laminar flow have mainly been performed through theoretical or numerical simulations, as demonstrated by Lee et al.^8^, Ems et al.^9^, and Lauga and Stone^10^. However, the mechanism of drag reduction on superhydrophobic surfaces in turbulent flows in previous studies remains inconsistent. A series of experiments studied the effect of turbulent flow on the superhydrophobic surface in drag reduction. Watanabe et al.^11^ and Zhao et al.^12^ reported that the drag reduction in turbulent flow is insignificant, which contradicts the claim in one of the earliest computational studies of this surface performed by Min and Kim^13^ demonstrating that the decrease in wall shear stress with increasing slip length, both perpendicular and parallel to the flow direction, leads to drag reduction in the flow. Recently, Martell et al.^14^ performed a direct numerical simulation (DNS) to investigate the drag reduction performance of the SHS in turbulent channel flow in combination with other surface geometries. Park and Sun^15^ explained that the discrepancy between experimental and simulation outcomes could be attributed to various factors. These factors encompass the loss of air within the trap, distortion of the air–water interface, and inaccuracies in drag force measurement. The cavity was used as an air trap and the interfacial behavior between air and water was investigated. However, during experimentation, instances occurred where certain amounts of air were lost from these traps, and they study also suggested that this loss of air has the potential to influence the characteristics of the air–water interface, leading to deviations from the anticipated results. Additionally, the air–water interface refers to the boundary or surface between the air and water phases, which was observed under different conditions or situations. However, during testing, the interface may become distorted. Factors such as changes in pressure, flow rate, or the presence of other forces can cause the interface to deform or change shape. These distortions can affect the measurements and interpretation of results, leading to potential errors results. Furthermore, frictional resistance is a force that occurs due to the interaction between a liquid (in this case, water), and a solid surface. Park and Sun^15^ investigated frictional drag on the air–water interface. However, accurately measuring frictional resistance can be challenging. Various factors, such as equipment limitations, perturbation effects, and uncertainties in pressure measurement, can cause errors in calculations. These errors can affect the reported values of frictional resistance, and subsequently influence the conclusions. Many simulation studies have been conducted to understand the mechanism of drag reduction by superhydrophobic surfaces (SHS) in turbulent flow. For example, Seo et al.^16^ studied the pressure loads on the air–water interface to understand the relationship between the size of textures and turbulent flow. In this study, the wall pressure statistics are divided into two components: coherent and time-dependent ones. The first is caused by the stagnation of slip flow at the solid posts, and the latter is by overlying turbulence. Larger SHS texture intensifies the stagnation pressure components, while turbulence remains insensitive to texture size. The stagnation pressure distribution and wall-normal pressure statistics are self-similar for different texture size. Their study analyzes the scaling of induced pressure and deformations of the air–water interface. The upper wavelength bound of the texture is quantified, limiting the robust operation of superhydrophobic surfaces in high-speed flows. Seo and Mani^6^ explored the impact of texture distribution on drag reduction. They found that the maximum interface deformation at the leading edge of the aligned distribution is twice as large as that observed in the case of randomly textures surface. Martell et al.^7^ investigated the effect of the texture size on drag reduction through a turbulent flow channel on an SHS, and found that increasing feature distance and free surface area increased slip velocity while reducing drag. The average shear velocity was above 80% of the obtained bulk velocity, and reduced the shear stress of the wall by greater than 50% at the largest micro feature spacing, and by increasing the feature distance and free surface area. Nguyen et al.^5^ reported that an aligned distribution with post shape can decrease drag reduction by 42%, restating the effect of texture on drag reduction in turbulent flow over an SHS. A few studies have also investigated the effect of the air layer on SHS, with Jung et al.^17^ investigating the effects of air-layer thickness of an idealized superhydrophobic surface that does not contain the texture on the surface by increasing air-layer thickness on slip length and skin-friction drag. They found that increasing air-layer thickness increased slip length, slip velocity, and drag reduction rate, as the shear flow driven by the air-layer provided larger slip than the recirculating flow. Alamé and Mahesh^18^ studied channel flow over a superhydrophobic surface with varying interface heights, and observed a nonlinear relationship between slip and drag reduction (DR) in the laminar flow case. In turbulent flow, they compared statistics with smooth walls, revealing a competing effect between the air–water interface and asperities on turbulence. The presence of trapped air alters the near-wall flow physics. However, most studies have ignored the effect of the air layer on drag reduction, considering it to be insignificant.

The present study focuses on exploring the impact of the air layer on flow resistance reduction in turbulent channel flow over a SHS. To achieve this, we examine the intricate coupling between the air–water surface, where the air layer plays a crucial role in modulating the flow characteristics. Turbulent flows are known for the complex and chaotic nature, and understanding how the air layer interacts with the water interface becomes essential in harnessing its potential for drag reduction. Through this coupling of air and water layers, we aim to shed light on the underlying mechanisms that govern the reduction of flow resistance in turbulent conditions. The presence of the air layer can significantly alter the flow dynamics, affecting parameters such as slip velocity and turbulent intensities. Consequently, this alteration may lead to a reduction in drag, providing potential applications in various industries where minimizing resistance and energy consumption are critical. We compare the present results with those from previous studies by using the near-wall velocity profiles and Reynolds stress profiles obtained without solving the air layer. The analysis of the coherent structures can be based on numerical simulation data. This study is one of a series of investigations on the characteristics and drag reduction mechanism of turbulent channel flow over SHS flow in our previous work ^5^. The results clarify whether the air layer has an effect on drag reduction in the channel flow over the SHS. In the following, section two briefly describes the governing equations, as well as the numerical model employed in the present study and simulation domain, while section three discusses them. Finally, Section four summarizes the main aspects of the present study.

## Computation model and simulation condition

### Contact state of superhydrophobic

**Figure 1:**
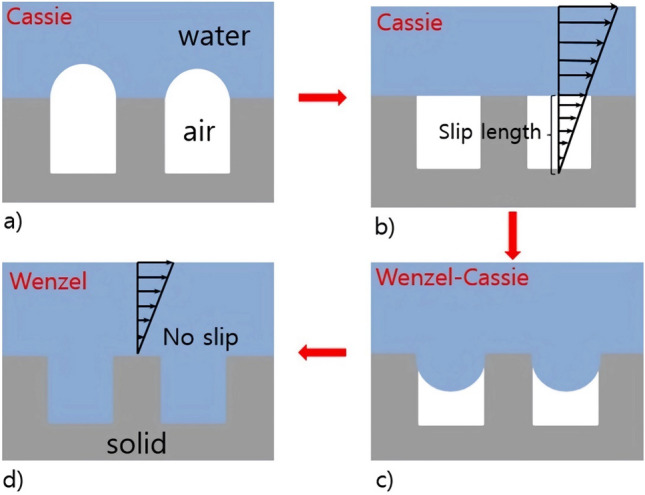
The contact state of SHS. (**a**) Depinned-out state, (**b**) pinned state, (**c**) pinned-in state, (**d**) fully wetted sate.

When the water flow on the surface is slow, resulting in a small pressure difference at the interface and hence a small or negligible contact angle, the interface assumes a convex meniscus state known as the Cassie state. In this state, surface tension prevents water from easily infiltrating the microstructural voids, thereby trapping air, as depicted in Fig. 1a. As the flow rate increases, the drag reduction also increases, leading to an increase in the pressure difference at the interface. When the water pressure equals the surface tension (Fig. 1b), the air–water angle becomes zero, reaching maximum drag reduction and slip length. Subsequently, the liquid gradually occupies space, transitioning to the Wenzel-Cassie state (Fig. 1c). With further increase in flow speed, the water pressure surpasses the surface tension, resulting in a change in the interface contact angle to a concave meniscus state. In this scenario, the rate of drag reduction slows down, and to a certain extent, the liquid can penetrate completely into the microstructural voids (Fig. 1d). This state is termed the Wenzel state, and the sliding velocity diminishes, leading to the loss of surface drag reduction.

**Figure 2:**
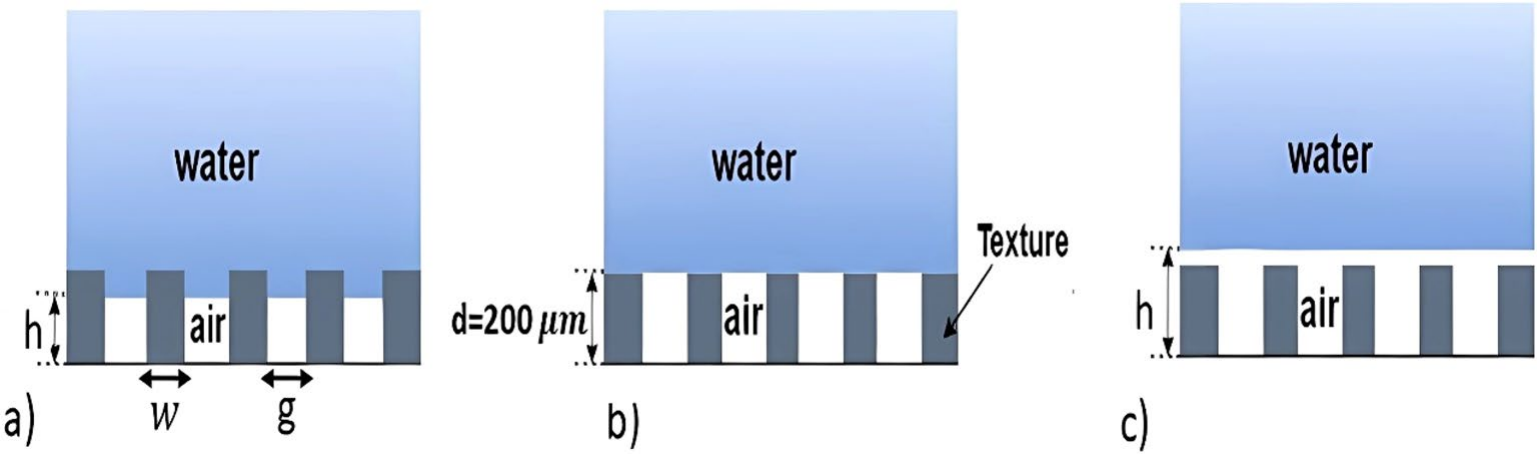
Schematics of the texture height and air layer: (**a**) Case 1, (**b**) Case 2, (**c**) Case 3.

For the purpose of this study, we will disregard the impact of surface tension by considering a flat at air–water interface. Our investigation will specifically focus on three states: Pinned-in state, Pinned state, and Depinned state. These correspond to Case 1, Case 2, and Case 3, respectively, as illustrated in Fig. 2.

In this study, we perform a direct numerical simulation (DNS) of a turbulent channel flow with an air layer over a super-hydrophobic surface (SHS). The SHS made from numerous microstructure textures with a post shape has been used in many previous studies, such as Martell et al.^7^, Seo and Mani^6^, and Nguyen et al.^5^. The size of the textures is 187.5 µm, and the distribution of textures is an aligned distribution that is referenced from past studies. Three cases with different height of the interface between water and air in the channel flow (from 140 to 260 µm) over the superhydrophobic surface are considered in the present work. Figure 2 shows the schematics of the three different cases. The height of the texture is *d* = 200μm as depicted in Fig. 2, a common size found in experiments^19,20^. We use the fluid–fluid coupling technique to simulate the behavior of both air and water. The water flow is simulated as an incompressible plane flow at *Re*_*τ*_ = 180. In its starting, uncoupled state, the interface is considered with a slip boundary condition, and the texture has a no-slip boundary condition. On the coupling interface with the air layer, the boundary conditions of the air have the same boundary condition as the water flow. The water flow sends velocity and pressure gradient to the air layer, and receives pressure from the air layer.

### Governing equations and simulation condition

Direct Numerical Simulation (DNS) is performed for a fully developed turbulent channel flow with a constant pressure gradient, and is combined with fluid–fluid coupling technique to investigate the effects of the air-layer of channel flow over a SHS on drag reduction. The incompressible fluid flow in the channel results in a constant, the corresponding friction Reynolds number equal to $e_{\tau } = \frac{{u_{\tau } \delta }}{{\nu_{water} }} = 180$, where *δ* is the half of channel height, the kinematic viscosity of the water $\nu_{water} = 1 \times 10^{ - 6} \,\left( {\frac{{{\text{m}}^{2} }}{{\text{s}}}} \right)$, and the wall-shear velocity $u_{\tau } = \sqrt {\frac{{\tau_{w} }}{\rho }}$. The wall shear stress, $\tau_{w} =\upmu \left( {\frac{\partial u}{{\partial y}}} \right)_{w}$ is calculated using the derivative in the normal direction of $\left\langle u \right\rangle$, in which the mean velocity value with respect to time (*t*) and double periodic in streamwise and spanwise direction of the channel (*x* and *z*). The initial velocity field of the simulation is generated based on the turbulent solution superimposed with a local random disturbance, which satisfies the continuity equation. The typical near-wall streaks are unstable to sinusoidal perturbations. The instability of the vortex-less streaks generates a base flow of the following form of Eq. (1)^21^ for three the velocity components:

1 $$u = U_{0}^{ + } \left( {y^{ + } } \right) + \left( {\frac{{\Delta u_{0}^{ + } }}{2}} \right)\cos \left( {b^{ + } z^{ + } } \right)\left( {\frac{{y^{ + } }}{30}} \right)e^{{\left( { - C_{1} y^{ + 2} + 0.5} \right)}}$$

2 $$v = 0$$

3 $$\omega = c_{\epsilon} \sin \left( {a^{ + } x^{ + } } \right)y^{ + } e^{{\left( { - c_{\sigma } y^{ + 2} } \right)}}$$

where $U_{0}^{ + }$ represents the mean bulk velocity of channel flow, which is defined by $U_{0} = \frac{{U_{0}^{ + } }}{{u_{\tau } }}$. $\Delta u_{0}^{ + }$ is the streak wall normal circulation, *b*^+^ the spanwise wavenumber, *c*_*σ*_ the transverse decay, $c_{\epsilon}$ the linear perturbation amplitude, *a*^+^ the *x*-wavenumber of the perturbation, *x*^+^, *z*^+^ and *y*^+^ presents for the treamwise spacing, the spanwise wavelength, and the height of the first mesh cell of the wall, respectively.

The governing equations for incompressible fluid flow are expressed in the Navier–Stokes equations^22^:

4 $$\frac{{\partial u_{i} }}{{\partial x_{i} }} = 0$$

5 $$\rho_{\varphi } \left( {\frac{{\partial u_{i} }}{\partial t} + \frac{\partial }{{\partial x_{j} }}u_{i} u_{j} } \right) = - \frac{\partial p}{{\partial x_{i} }} + \mu_{\varphi } \frac{{\partial^{2} u_{i} }}{{\partial x_{j} \partial x_{j} }} + \Pi_{\varphi } \delta_{1i}$$

where *t* is the time, *x*_*i*_ the coordinate of the streamwise, wall-normal, and spanwise direction, corresponding to $\left( {x_{1,} x_{2} ,x_{3} } \right) = \left( {x,y,z} \right)$, and (*u*_1_, *u*_2_, *u*_3_) = (*u*, *v*, *w*) the velocity components, p the pressure, *ρ*_*φ*_ and *μ*_*φ*_ the density and viscosity of water or air, respectively ($\rho_{water} = 1000\,\left( {\frac{{{\text{kg}}}}{{{\text{m}}^{{3}} }}} \right)$, $\rho_{air} { } = { }1.2\,\left( {\frac{{{\text{kg}}}}{{{\text{m}}^{{3}} }}} \right)$, *μ*_*water*_ = 1.0 × 10^−3^ Pa s, *μ*_*air*_ = 1.8 × 10^−5^ Pa s at the standard condition: 20 °C and 1 atm), Π_water_ the constant pressure gradient necessary to drive the water flow, and Π_air_ the forcing term to determine the air-layer flow.

**Figure 3:**
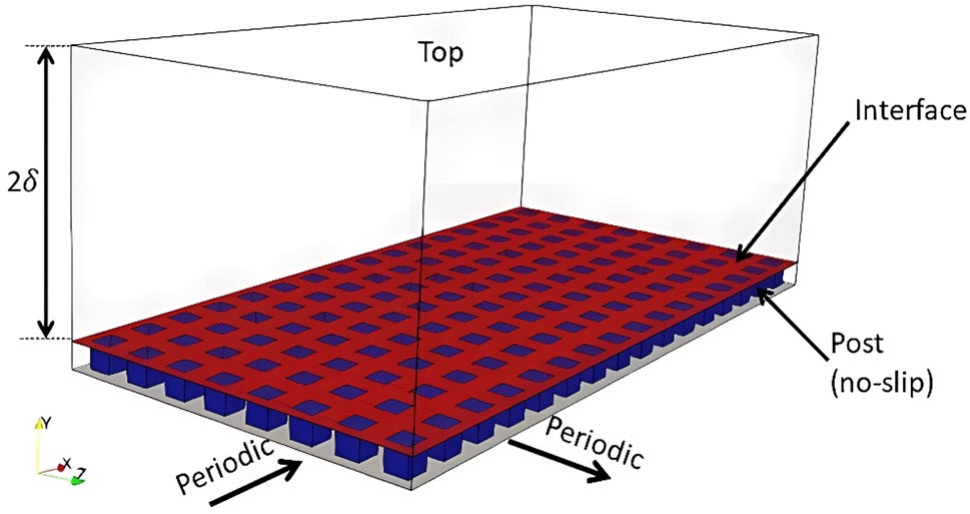
Schematic of the geometry and boundary condition for the simulation (Case 1).

**Figure 4:**
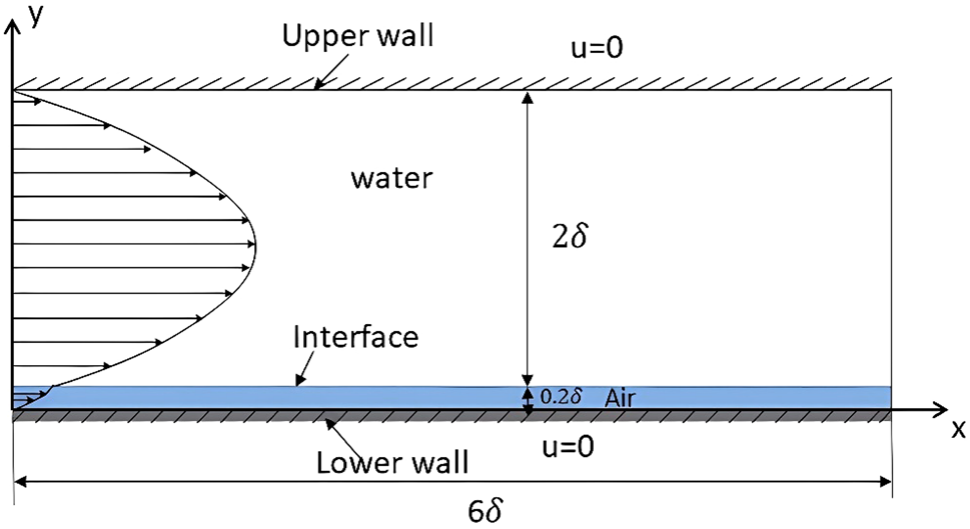
Schematic of the computational domain (xy plane) of the simulation.

In this study, the open-source Computational Fluid Dynamics (CFD) tool, OpenFOAM^23^, which is based on the finite volume method, is adopted for the fluid solver. OpenFOAM has been widely used for Direct Numerical Simulation (DNS) for turbulent channel flow in various studies^24–27^, and the results have been compared with simulation results in turbulent channel flow with high order method^22^, and the results from experimental data^28^. To discretize the temporal derivative, the second-order implicit backward scheme is adopted. The usual pressure-implicit split-operator (PISO) algorithm^29–31^, which is a popular and robust numerical scheme for the pressure–velocity coupled equation, is considered. The periodic boundary conditions are applied to the streamwise and spanwise directions of the channel and no-slip boundary conditions on the solid surface at the upper (top wall) and lower (bottom wall) surface and the surface of the posts, while the water–air interface is assumed to be flat, the wall will have coupled velocities and pressure to be continuously maintained across the interface, which is illustrated in Figs. 3 and 4.

**Figure 5:**
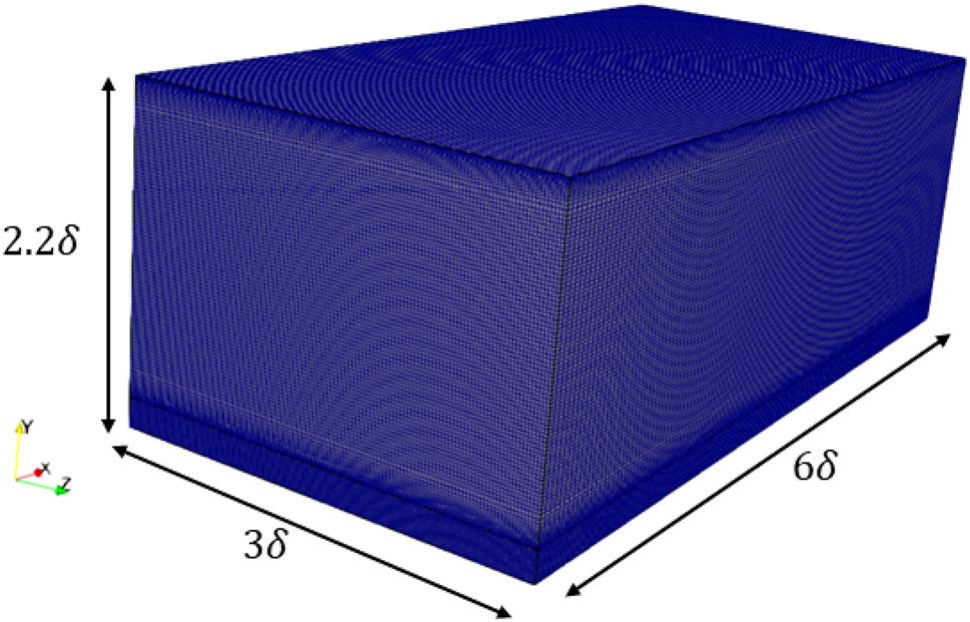
Schematic of the mesh.

The dimensions of the computational domain were 6δ × 2.2δ × 3δ in the streamwise, wall-normal, and spanwise directions, respectively. A uniform mesh is applied in the streamwise and spanwise directions, with grid spacing of Δ*x*^+^ ≈ 7.0, and Δ*z*^+^ ≈ 5.0 in wall unit, respectively. For the wall-normal direction, a non-uniform mesh is used, with minimum sizes of $\Delta y_{min}^{ + } \approx 0.3$ and maximum sizes of $\Delta y_{max}^{ + } \approx 3.0$, corresponding to the number of mesh 128 × 128 × 128, which are sufficient to simulate fully developed turbulent flow in DNS ^22,32^, as seen in the schematic of Fig. 5 and Table 1. The Courant–Friedrich–Lewy (CFL)^33^ number is calculated based on $\left\{ {\overline{{u_{x} }} } \right\}_{{y^{ + } }}$ is *U*_*bulk*_: CFL ≈ 0.3 with the time step size $\Delta_{t} = 1 \times 10^{ - 6} \,{\text{s}}$. The linear equation system for the velocity is solved by Gauss–Seidel Smoother (GSS)^23^ solver, while for the pressure, Geometric–algebraic multi-grid (GAMG)^23^ solver is applied to the linear equation system. The simulations were conducted in the supercomputer (HPC) facilities of the Korea Institute of Science and Technology Information (KISTI). The simulation was parallelized with Message Passing Interface (MPI), which provides 200 Intel Xeon processors and 2 TB of central memory. The simulations were conducted using an averaging strategy. The cases were integrated for up to 10 eddy turn-over times ($t = 10\frac{\delta }{{u_{\tau } }}$) from random turbulent initial conditions^34^. Once the initial transients dissipated, statistics were gathered for several eddy turn-over times until the flow reached statistical stationary which is T = 500t (total time to run simulation), as shown in Table 1.

**Table 1:** Parameters of numerical configuration, and with the results of centerline value for DNS channel flow at *Re*_*τ*_ = 180.

Database	*Re*_*τ*_	Domain			Grid			Resolution			T	Centerline value			
		*L*_*x*_	*L*_*y*_	*L*_*z*_	*N*_*x*_	*N*_*y*_	*N*_*z*_	Δ*x*^+^	$\Delta y_{min}^{ + }$	Δ*z*^+^		*U*_*bulk*_	*u*_*rms*_	*v*_*rms*_	*ω*_*rms*_
Kim et al. ^27^	178.1	3*πδ*	2*δ*	*πδ*	128	128	128	17.7	0.5	5.9	?	18.3	0.814	0.612	0.589
Present study	180	6*δ*	2.2*δ*	3*δ*	128	128	128	7.0	0.3	5.0	500	18.4	0.795	0.615	0.610

### Two-way coupling method at interface

**Figure 6:**
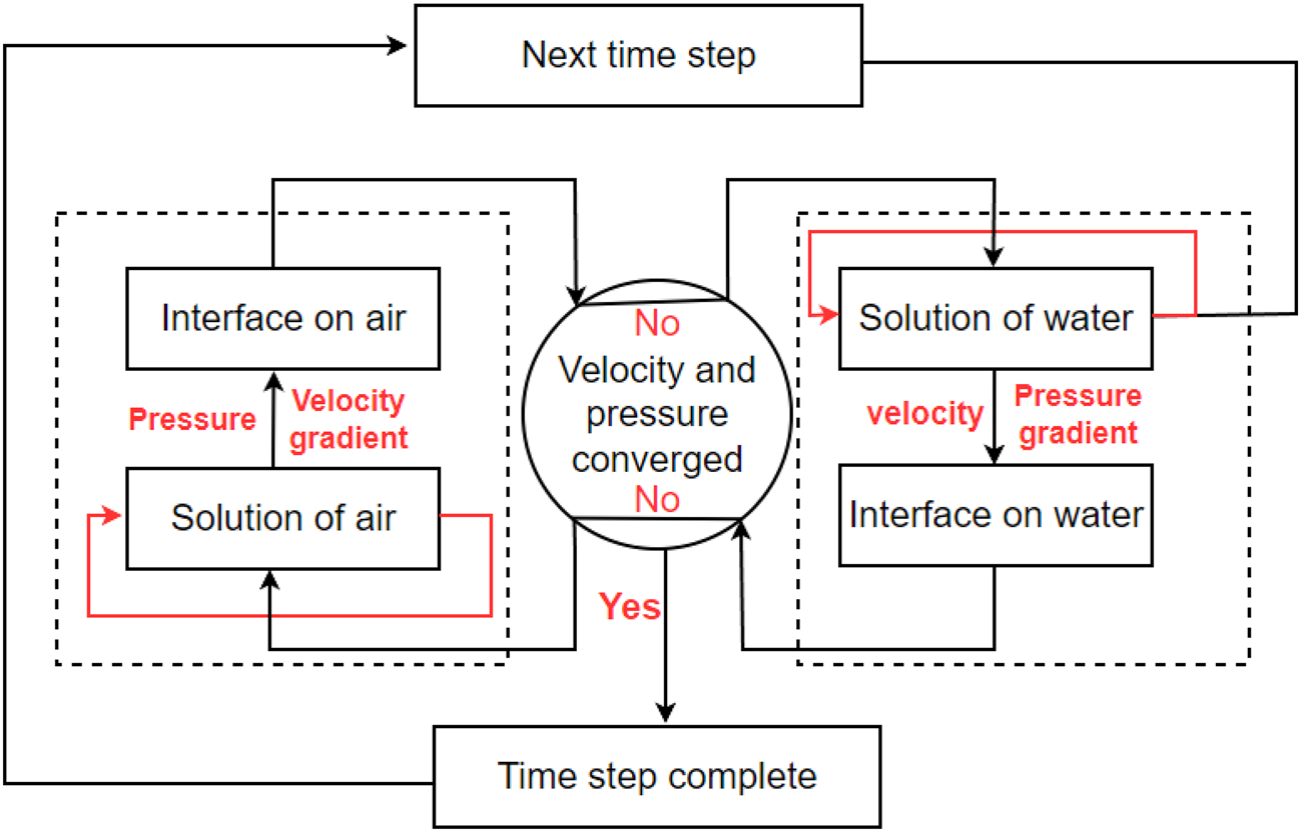
Solution algorithm of two-way coupling for water and air.

As mentioned in previous session 2.1, the surface tension force can be neglected with increase of the flow rate. This implies that there will be no deformation at the interface. Therefore, the fluid–fluid coupling method is applied with assumption of flat interface. For the fluid–fluid coupling between water and air flow, preCICE software^35^, which is an open-source library coupled for partitioned multi-physics simulations, is adopted. The fluid–fluid coupling module provides shared variables as velocity, pressure, and their gradients for partitioned flow simulation. The technique was previously used by Chourdakis et al.^36^, and has been shown to be effective for reproducing multi-model based flow simulations, such as the water hammer example. The technique works by allowing one fluid flow to send velocity and pressure gradient values to another fluid flow at the interface, and vice versa. This creates a continuous exchange of information between the two fluids, and enables accurate modeling of the fluid flow behavior at the interface. In this study, the interface between water and air serves as the boundary between the two fluid flows. The velocities and pressures are maintained to be continuous across the interface. Equations (6)–10) represent the mathematical framework used to ensure continuity at the interface, and their significance lies in the ability to accurately model the fluid flow behavior at the water–air interface. The overall structure of the solution algorithm for this simulation is represented in Fig. 6:

6 $$u_{water,s} = u_{air,s} ,\quad w_{water,s} = w_{air,s}$$

7 $$P_{water,s} = P_{air,s}$$

8 $$\left. {\frac{{\partial u_{water} }}{\partial y}} \right|_{s} = \left. {\frac{{\partial u_{air} }}{\partial y}} \right|_{s} ,\quad \left. {\frac{{\partial w_{water} }}{\partial y}} \right|_{s} = \left. {\frac{{\partial w_{air} }}{\partial y}} \right|_{s}$$

9 $$\left. {\frac{{\partial P_{water} }}{\partial y}} \right|_{s} = \left. {\frac{{\partial P_{air} }}{\partial y}} \right|_{s}$$

10 $$v_{s} = 0$$

where the subscript ‘s’ is the value at the interface. The wall-normal velocity at the interface is zero to satisfy an impermeability condition:

Figure 6 illustrates the process flow chart for the two-way coupling of water and air. Initially, the water field is solved until convergence criterion is achieved (these results are taken as results for simulation cases without considering the air layer). The resulting velocity and pressure gradient at the interface are then transferred to the air side. Subsequently, the air side incorporates these values (velocity and pressure gradient) as boundary conditions for solving the air field equations, as outlined in Eqs. (6 and 9). The air field is then solved to compute the pressure and velocity gradient (Eqs. 7 and 8) at the interface, and these values are transferred back to the water side. This sequence completes one iteration of the simulation's inner loop. These steps are iteratively performed until changes in velocity and pressure drop below a predefined threshold (the loop is twenty or the error value is lower than 10^−4^). Following this, a new time step is initiated. The solver used for the water and air regions is exactly the same as presented above.

**Figure 7:**
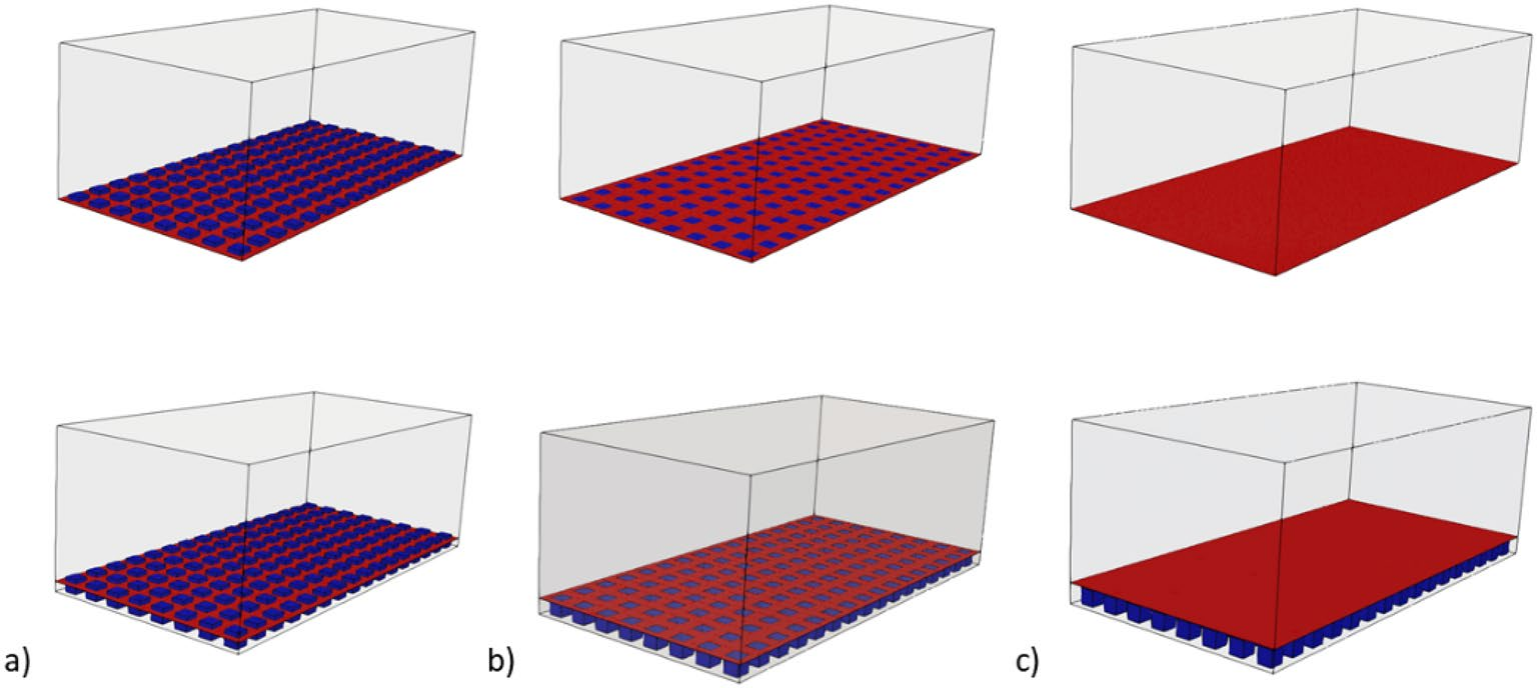
3D Schematics of simulation (**a**) Case 1, (**b**) Case 2, (**c**) Case 3: with air-layer (bottom) and without air-layer (top).

The purpose of this study is to investigate the effects of varying air layer heights on the behavior of a flat meniscus on a textured surface. The study explores three different cases with different air layer heights. In Case 1, the air layer height is lower than the height of the texture, leading to a flat meniscus that is displaced into the structures (Pinned-in state). In Case 2, the air layer height is equal to the height of the texture, resulting in a flat meniscus that does not displace (Pinned state). In Case 3, the air layer height is higher than the height of the texture, causing the flat meniscus to be displaced off the structures (Depinned-out state). Furthermore, this study aims to investigate the role of air layer on the level of drag reduction of the SHS. The results obtained from the cases with air layer are compared to those obtained from cases without air layer, where a slip boundary condition is applied to the interface (as shown in Fig. 7). By quantifying the extent to which the presence of air layer contributes to drag reduction, this comparison provides insights into the underlying mechanisms involved in drag reduction flow.

In the present work, the solid fraction, $\emptyset_{s}$, is calculated using the equation $\emptyset_{s} = \frac{{w^{2} }}{{\left( {w + g} \right)^{2} }} = 0.25$, where *w* and *g* represent the width and gap in the SHS surface (see Fig. 2), respectively. The sizes of both width and gap are 187.5 µm^5–7^. This paper is a continuation of Nguyen et al.^5^, which explores the effect of the solid fraction and distribution shapes on the performance of the superhydrophobic surface. The solver used in this study was validated on the channel flow simulations with smooth wall by Kim et al.^22^.

**Figure 8:**
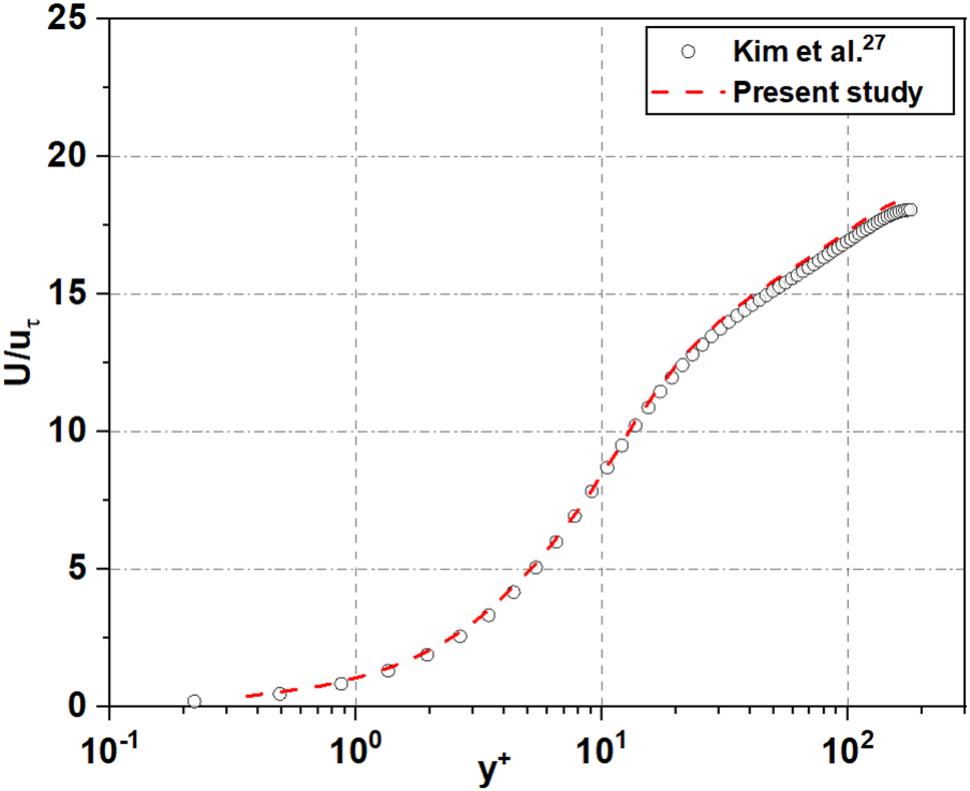
A comparison of wall velocity profiles for turbulent channel flow at *Re*_*τ*_ = 180.

**Figure 9:**
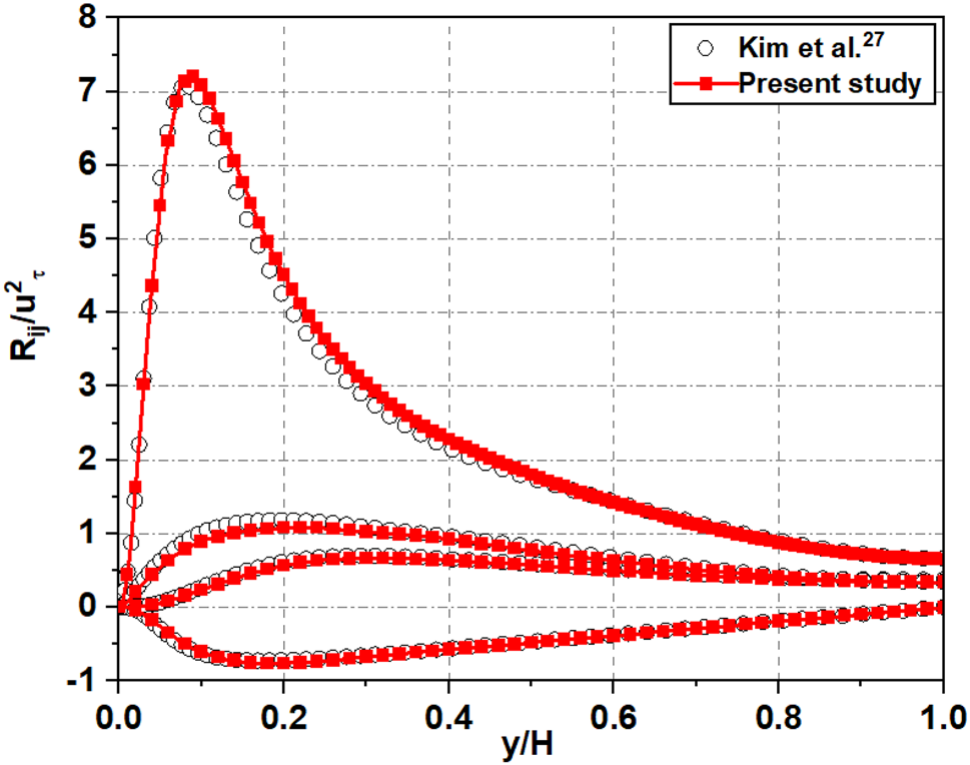
A comparison of Reynolds stress profiles (*R*_*ij*_) for turbulent channel flow at *Re*_*τ*_ = 180.

**Figure 10:**
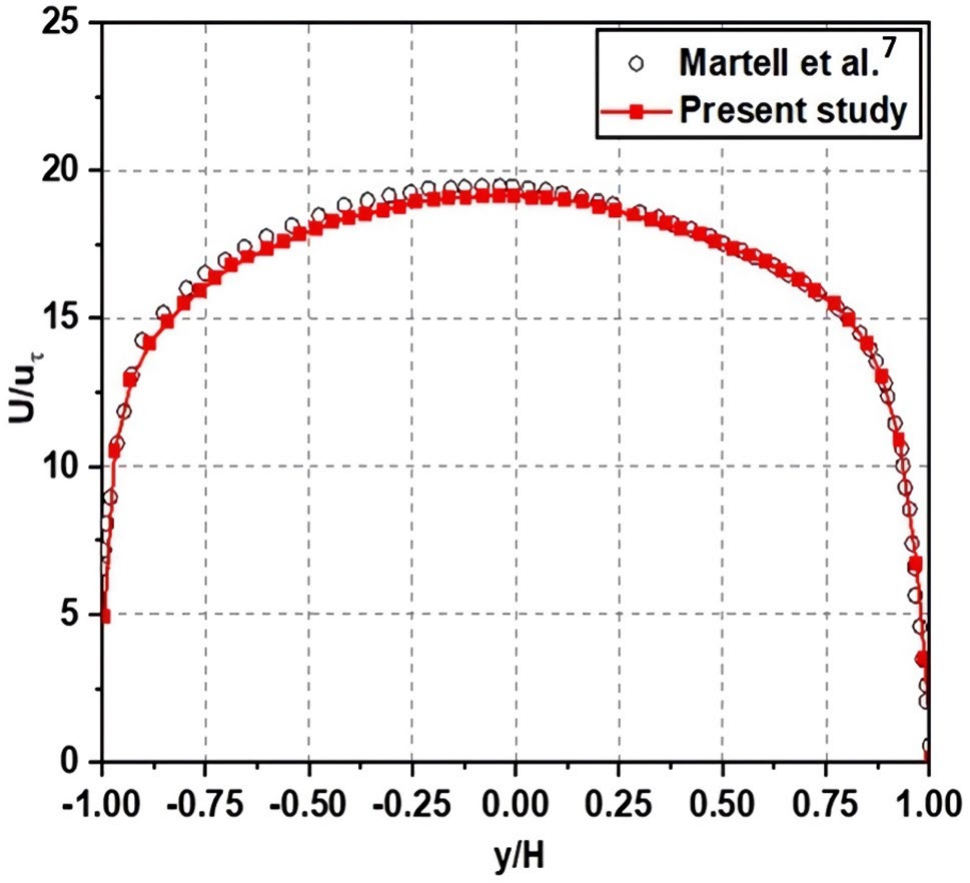
A comparison of the mean streamwise velocity profiles for channel flow with SHS.

As shown in Figs. 8 and 9, first, the standard channel flow with the solid walls was validated at the condition of *Re*_*τ*_ = 180. Additionally, to ensure that it also works correctly with a channel flow featuring a superhydrophobic surface, we conducted a comparison with Martel et al.’s results at *Re*_*τ*_ = 180 and $\emptyset_{s}$ = 0.25^7^, which had the same boundary conditions and the solid fraction rate and with the same ridge shape for texture for SHS. This comparison^5^ allows the accuracy and effectiveness of the adopted solver to be validated in the present work under a different flow scenario. We compare the mean streamwise velocity profiles of the flow where the velocity is normalized by *u*_*τ*_ of the water at the interface, as show in Fig. 10. The results are encouraging, as they indicate similar shifts in peak velocity towards a superhydrophobic surface. Thus, it can be confirmed that the present calculation is reliable for the prediction of statistical quantities of the superhydrophobic surface turbulent channel flows:

## Results and discussion

### Analysis of slip quantities and drag reduction

**Figure 11:**
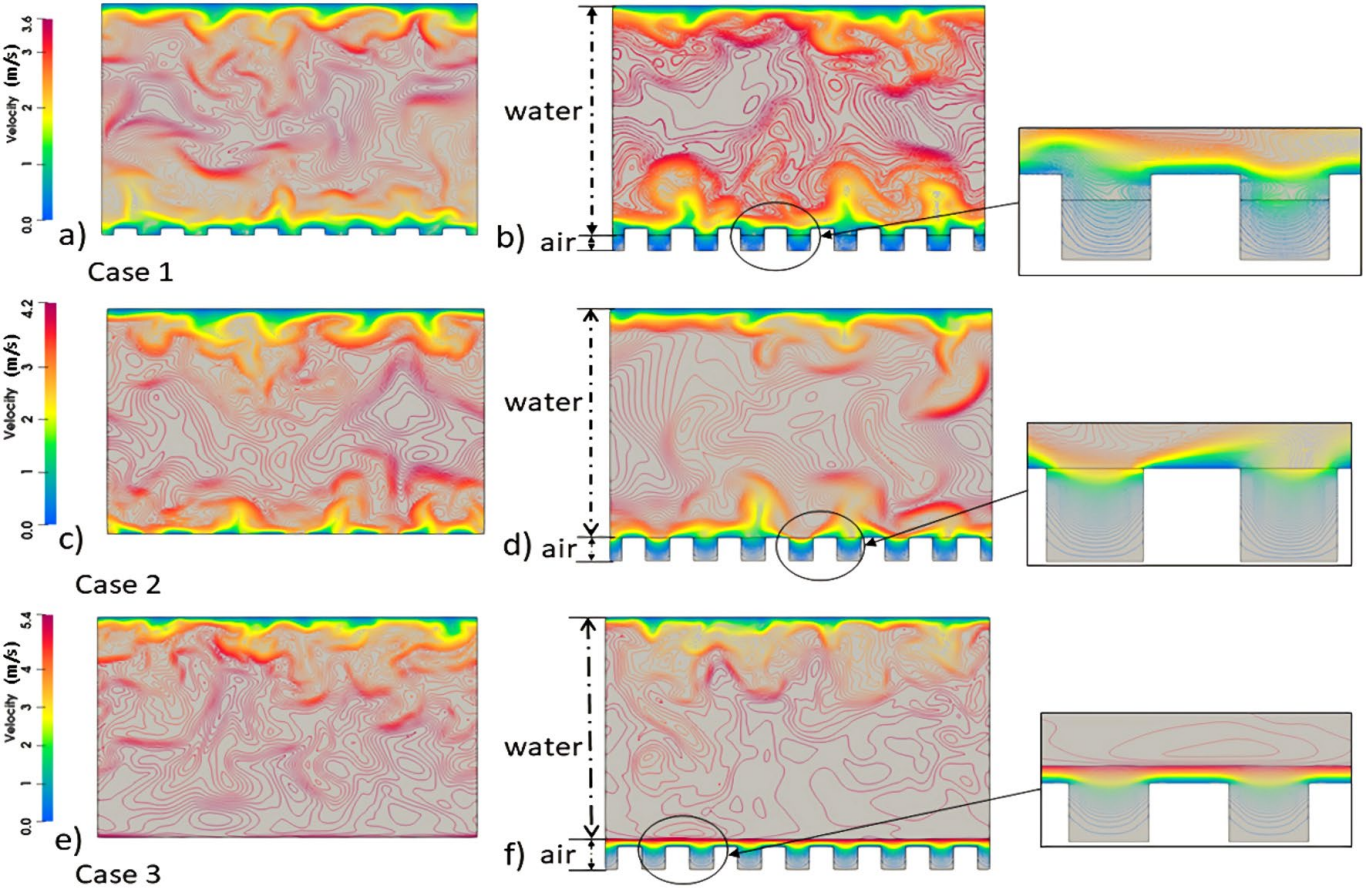
The contour of instantaneous velocity magnitude (the left column: cases without air layer, the right column: cases with air layer).

Figure 11 illustrates the contour of the instantaneous velocity magnitude through the yz plane, enabling a comparison of velocity distributions between the cases with and without an air layer. In the simulation results of the cases involving air layer, the contours of the instantaneous velocity demonstrate continuity across the interface separating the water and air. This observation serves as a confirmation that the coupling solver functions effectively, faithfully reproducing the values at the interface. The accurate inheritance of values at the interface aligns precisely with the initial assumption for this study, which aims to examine the impact of the air layer on drag reduction. By maintaining the integrity of the velocity profiles through the interface to ensure that the subsequent analysis can reliably assess the effect of the air layer on drag reduction.

**Figure 12:**
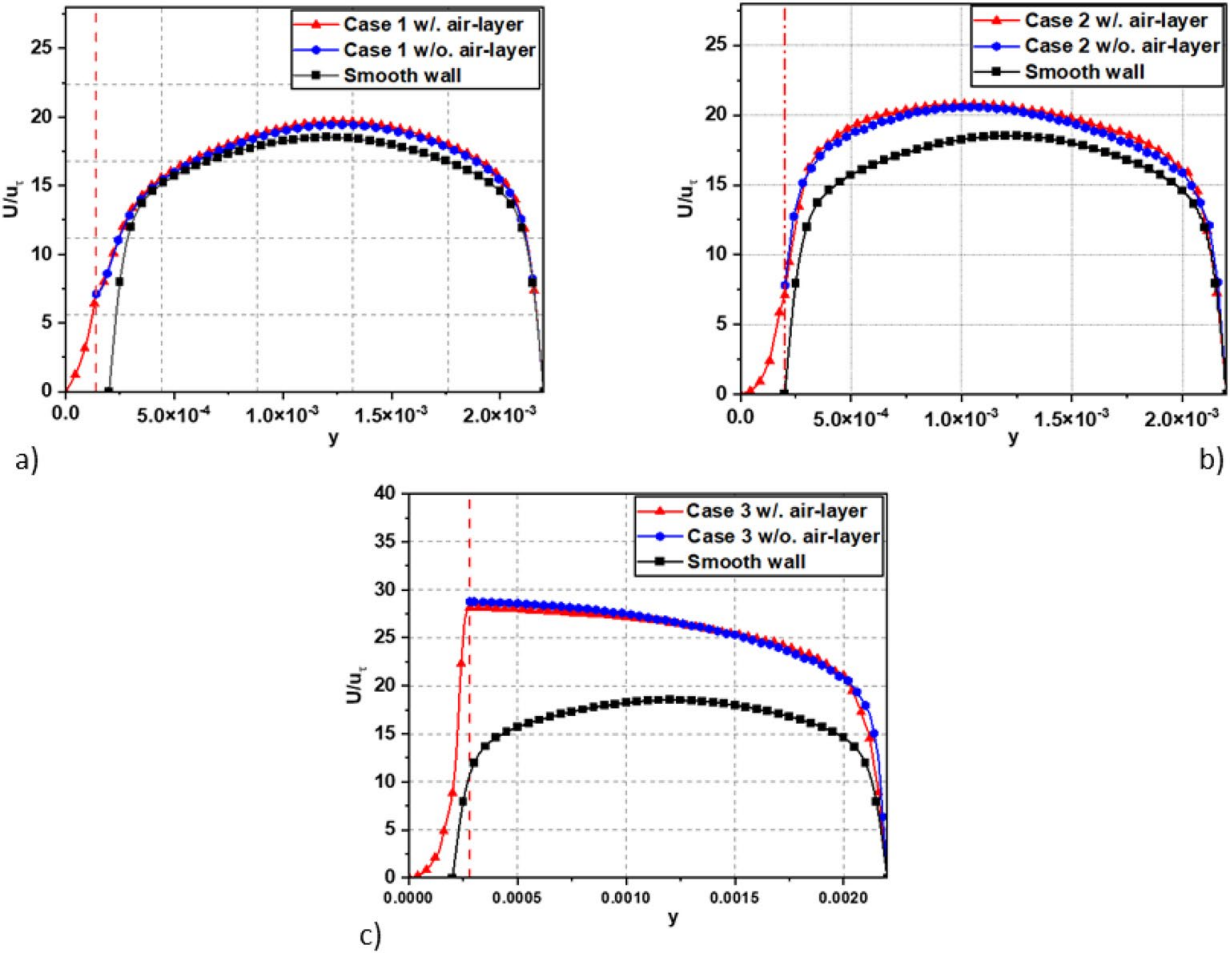
The velocity profiles are shown for the investigated cases: (**a**) Case 1, (**b**) Case 2, and (**c**) Case 3. In simulations with the air layer, the red dot line represents the water–air interface, with the right side indicating the water region, and the left side indicating the air region.

**Figure 13:**
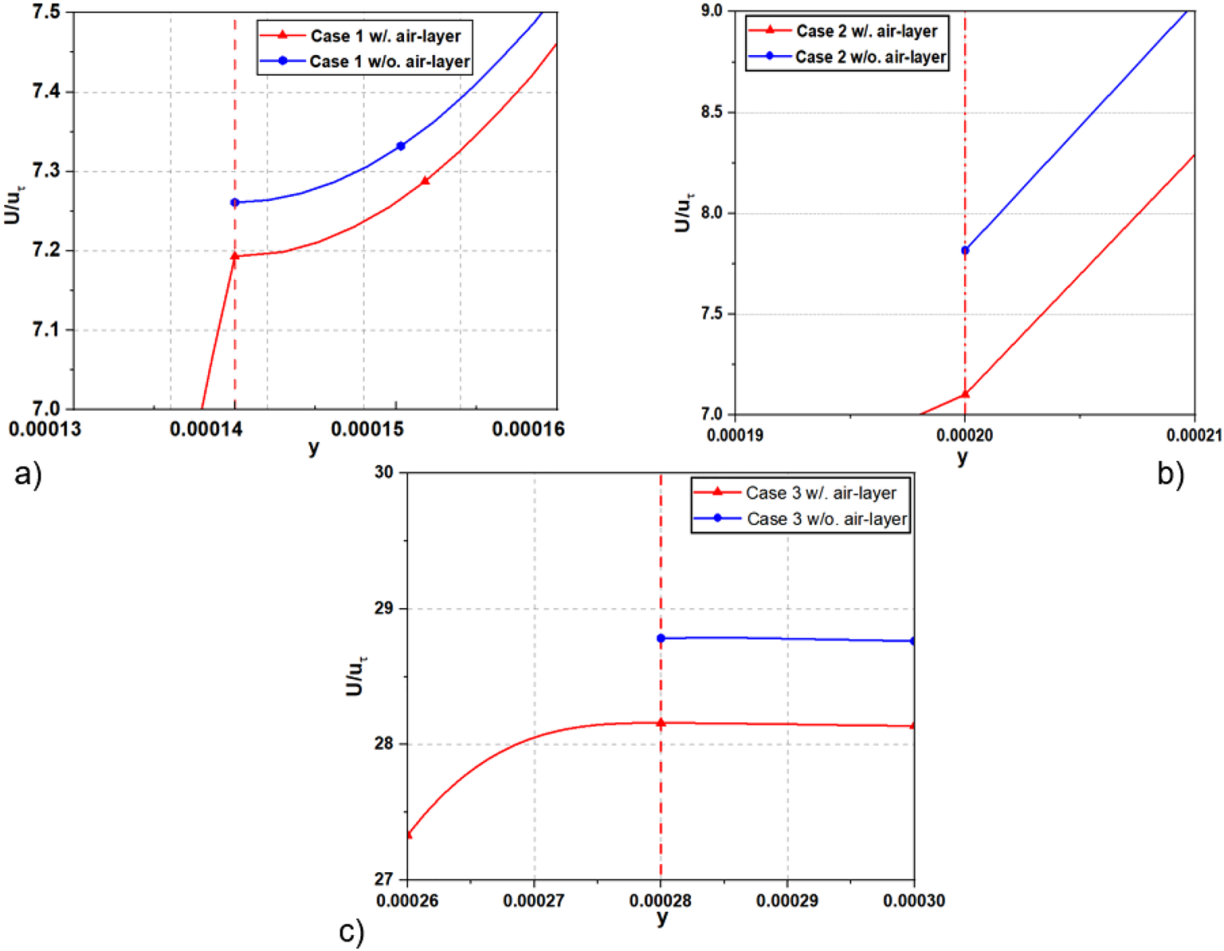
The local velocity profile at the interface for the investigated cases.

To investigate how an air affects drag reduction in channel flow, we conducted a comparative analysis of mean streamwise velocity profiles for two ways: one with an air layer, and the other without in Fig. 12. While Fig. 13 show the local velocity profile at interface for all investigated cases. The velocities are normalized by *u*_*τ*_ at the condition of *Re*_*τ*_ = 180. It is found that revealed that the presence of the air layer directly influences the velocity at the interface and bulk velocity, resulting in a change in the slip velocity, and leading to a change in the drag reduction of flow. The simulations with an air layer showed lower velocities at the interface compared to simulations without such a layer. This observation suggests that the presence of the air layer reduce flow dynamics near the surface, leading to reduced velocities when comparing simulations incorporating the air layer with those assuming the neglect of the air layer as a slip boundary condition, as presented in the paper and as postulated by previous studies^7,13,14,16^. This is explained by the reason that the velocity of the air layer at the interface can reduce the velocity of the liquid, causing a decrease in viscosity (slip) between the air and water layers. Consequently, this prompts the adoption of an assumed slip boundary condition at the interface during simulation. Contrary to this, in the case of assuming a slip boundary condition at the interface, the simulation presupposes the absence of an air layer, and the velocity of the liquid at the interface is determined based on slip conditions. This can result in no influence from the air layer, and thus, the velocity at the interface may remain unchanged or not decrease significantly, as in the case with the air layer.

**Figure 14:**
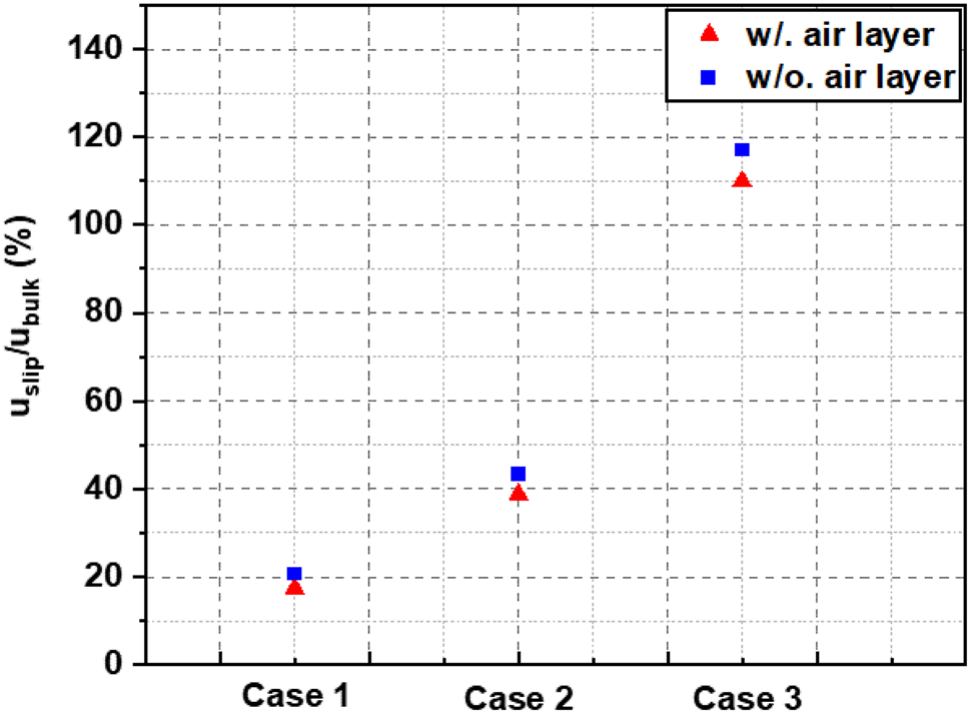
The slip velocity at interface normalized by *U*_*bulk*_.

Additionally, we investigated how the thickness of the air layer impacts the velocity at the interface surface. Our findings indicate a significant role of air layer thickness in determining the interface velocity. Specifically, an increase in air layer thickness corresponds to a proportional increase in interface velocity. This observed correlation implies that a greater thickness of the air layer enhances flow dynamics efficiency, resulting in higher velocities at the interface. Additionally, we can determine the slip velocity (*u*_*s*_) by examining the velocity at interface in the figures. The results of the present study shed light on the often-overlooked influence of the air layer on the velocity at the interface, a factor that has been disregarded or neglected in many previous studies^7,8,13,14,16,18^. Comparing results without an air layer to those with an air layer, we observed a difference in interface velocity ranging approximately from 3 to 7%. As the thickness of the air layer increases, this difference gradually increases, as shown in Fig. 14. Although the difference in velocities at the interface and the bulk velocities of the two simulations with and without the air layer is not large, these results highlight the importance of considering the air layer when analyzing velocity profiles near the interface. By accounting for the presence and thickness of the air layer, we can obtain more precise insights into the velocity distribution and its variations near the interface. Understanding these effects is crucial for designing and optimizing systems where drag reduction and flow efficiency are desired.

Calculation of the slip length (*λ*) is not a straightforward task. The definition of the slip length is the ratio between the slip velocity and the velocity gradient at the interface. Therefore, to accurately determine the slip length, we need to analyze the velocity gradient at the interface. The slip length is obtained from = $\frac{{\overline{{u_{s} }} }}{{\left( {\frac{{\partial \overline{{u_{w} }} }}{\partial y}} \right)_{s} }}$, where $\overline{u}_{s}$ is the mean slip velocity, and $\left( {\frac{{\partial \overline{{u_{w} }} }}{\partial y}} \right)_{s}$ is the mean velocity gradient of water at the interface. In the study, the average shear stress over the top and bottom walls is calculated by the equation presented in references^37–39^: $\tau_{\omega }^{t} + \tau_{\omega }^{b} = 2\rho {\text{G}}\delta$, where G, which is defined by $u_{\tau } = \sqrt {G\delta }$, is the prescribed pressure gradient. The $\tau_{\omega }^{t}$ and $\tau_{\omega }^{b}$ are the average shear stresses at the top with smooth wall and bottom SHS wall, respectively. As mentioned above, we can derive the average velocity gradient at the bottom wall in another way,$\left( {\frac{{\partial \overline{{u_{w} }} }}{\partial y}} \right)_{b} = \frac{2G\delta }{\nu } - \left( {\frac{{\partial \overline{{u_{w} }} }}{\partial y}} \right)_{t}$. Finally, the slip length at the SHS wall can be calculated as follows:

11 $$\lambda = \frac{{\overline{{u_{s} }} }}{{\frac{2G\delta }{\nu } - \left( {\frac{{\partial \overline{{u_{w} }} }}{\partial y}} \right)_{t} }},$$

Table 2 presents the slip length and slip velocity values for each survey case, showing the relationship between the air layer height and the slip velocity and the corresponding slip length. The data in Table 2 show that as the height of the air layer increases, the slip velocity and slip length change proportionally. In particular, the simulation results related to the *u*_*slip*_/*u*_*bulk*_ ratio in Case 2 are most similar to natural physical phenomena and are also the case that has been widely explored in many previous studies^6–8^ to study drag reduction mechanism of superhydrophobic surface (SHS). The rate *u*_*slip*_/*u*_*bulk*_ of 43.38% observed in Case 2 of this study is remarkably consistent with the findings reported by Martell et al.^7^ at the same SHS characteristics and *Re*_*τ*_ = 180 (without consider air layer). In Case 3, the ratio *u*_*slip*_/*u*_*bulk*_ is over 100% for both simulations, with or without an air layer. The maximum velocity occurs at the interface position, as depicted in Fig. 12c. This means that the fluid will have the ability to move quickly and smoothly across the surface, and its maximum velocity will appear at the surface. This setting is different from the boundary condition setting for Case 1 and Case 2 where the slip and no-slip boundary conditions alternate as shown in Fig. 7a,b. In the no-slip regions, the fluid experiences strong adhesion to the surface, creating a slow-moving layer close to the surface. On the other hand, in the slip regions, the fluid can move smoothly, accelerating, and generating a less significant slow-moving layer. As a result, the velocity profile will take on a parabolic shape as shown in Fig. 12a,b.

**Table 2:** Slip length and slip velocity for the investigated case at the DNS channel flow at *Re*_*τ*_ = 180 over SHS.

Case	g = w (μm)	h (μm)	slip length (λ) (μm)	*u*_*slip*_/*u*_*bulk*_ (%)
Case 1				
w/ air layer	187.5	140	0.18	16.84
w/o air layer	187.5	140	0.28	20.52
Case 2				
w/ air layer	187.5	200	68.3	38.324
w/o air layer	187.5	200	72.5	43.38
Case 3				
w/ air layer	187.5	260	301.5	109.68
w/o air layer	187.5	260	362.4	117.14

The superhydrophobic surface has shown significant potential in reducing drag at the water–air interface. Typically, the drag reduction is calculated by measuring the reduction in the required pressure gradient to deliver the same mass flow rate. However, the approach used by Martell et al.^7^ did not use a constant pressure gradient, rendering this definition invalid. Instead, they used the reduction of the wall shear stress at the superhydrophobic surface, normalized by the average wall shear stress. The present approach provides a more accurate representation of drag reduction in SHS by using Eq. (10)^40^; we can quantify the amount of drag reduction achieved by SHS:

12 $$DR\,\left( \% \right) = 100 \times \left[ {\frac{{C_{f,Basline} - C_{f, SHS} }}{{C_{f,Basline} }}} \right]$$

where *C*_*f*_ is the skin-friction coefficient, which is for the channel with smooth wall for both top and bottom wall, and *C*_*f*,*SHS*_ is used to denote the resistance coefficient for the SHS. We used them to determine the skin coefficient and the average local wall shear stress *τ*_*ω*_, as show in Eqs. (13) and (14):

13 $$C_{f} = \frac{{\tau_{\omega } }}{{\frac{1}{2}\rho U_{bulk}^{2} }}$$

14 $$\tau_{\omega } = - \delta \frac{\partial P}{{\partial x}} = - \delta G$$

**Figure 15:**
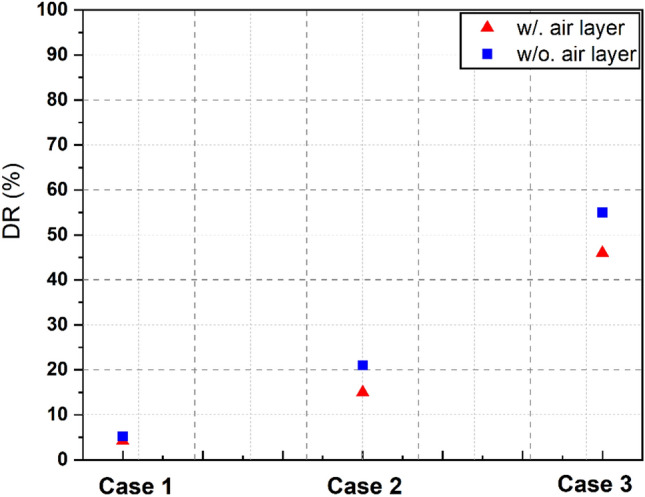
The drag reduction rate of the surveyed cases.

Figure 15 shows a tendency for the reduction to gradually increase as the air layer thickness increases, as expected. As the thickness of the air layer increases, the frictional drag decreases. The thicker the air layer, the more it has the potential to decrease the opposing drag force acting on the surface, thereby enhancing the capability to reduce drag in turbulent flow. Additionally, the results show that the simulation model with air layer has a lower drag reduction than the simulation without an air layer. While this difference is not substantial, it is approximately from 5 to 10%. The difference between the two models is the greatest in Case 3, in which the air layer thickness is the largest. This implies that the behavior of the velocity and pressure inside the air layer have a more effect on the interface and water side for the drag reduction as the air layer thickness increases.

### Analysis of the Reynolds stress

**Figure 16:**
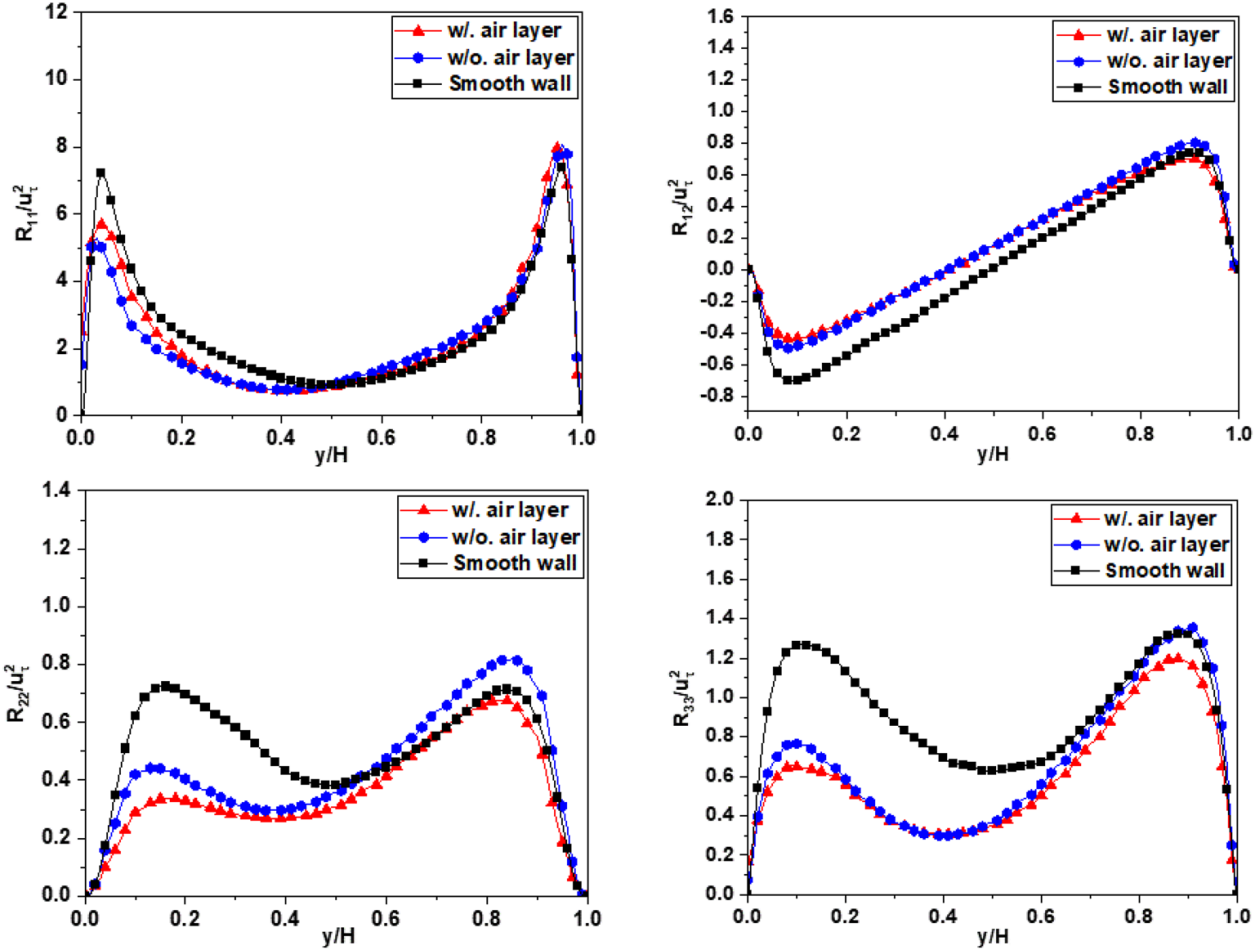
The Reynolds stress profile for Case 1.

**Figure 17:**
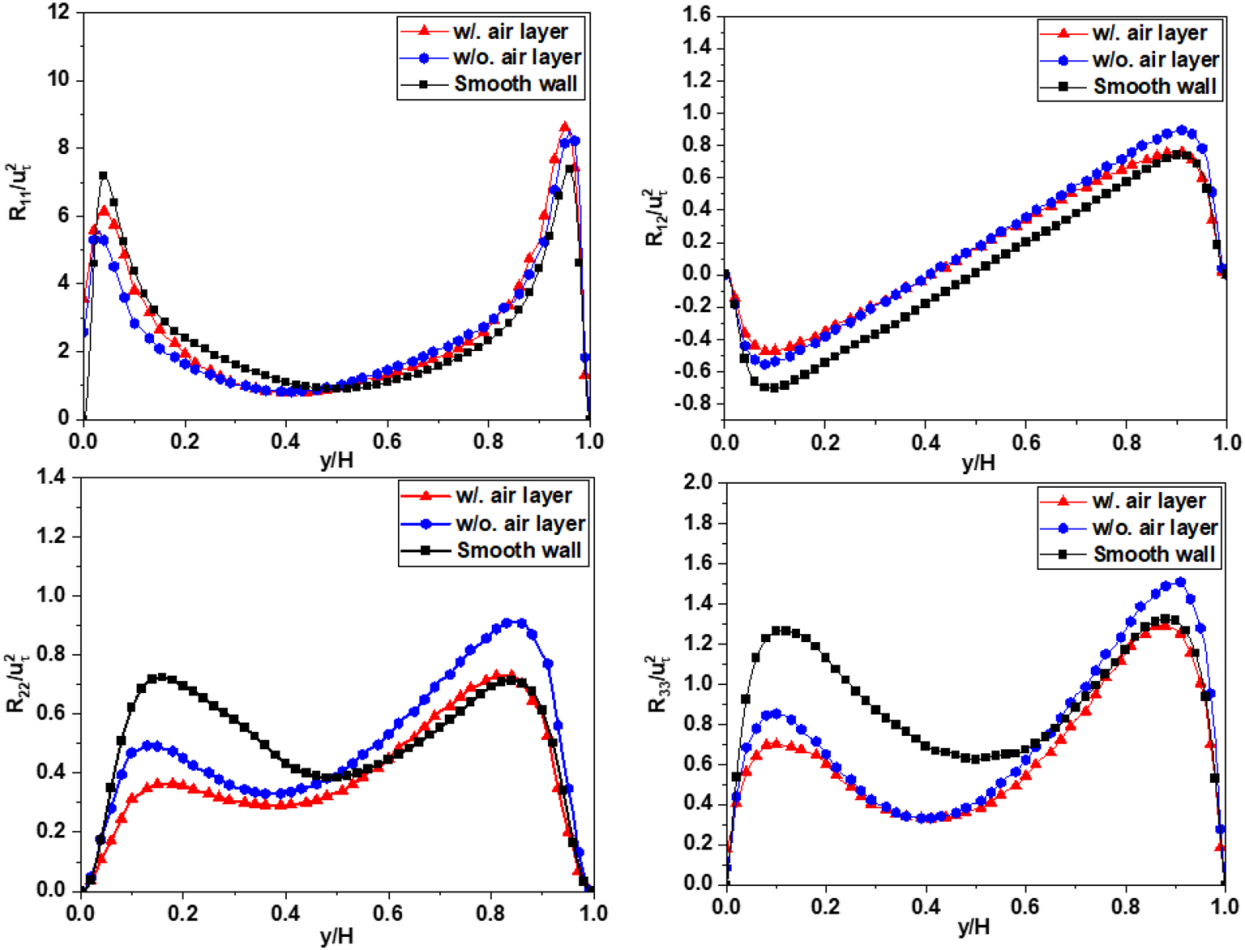
The Reynolds stress profile for Case 2.

**Figure 18:**
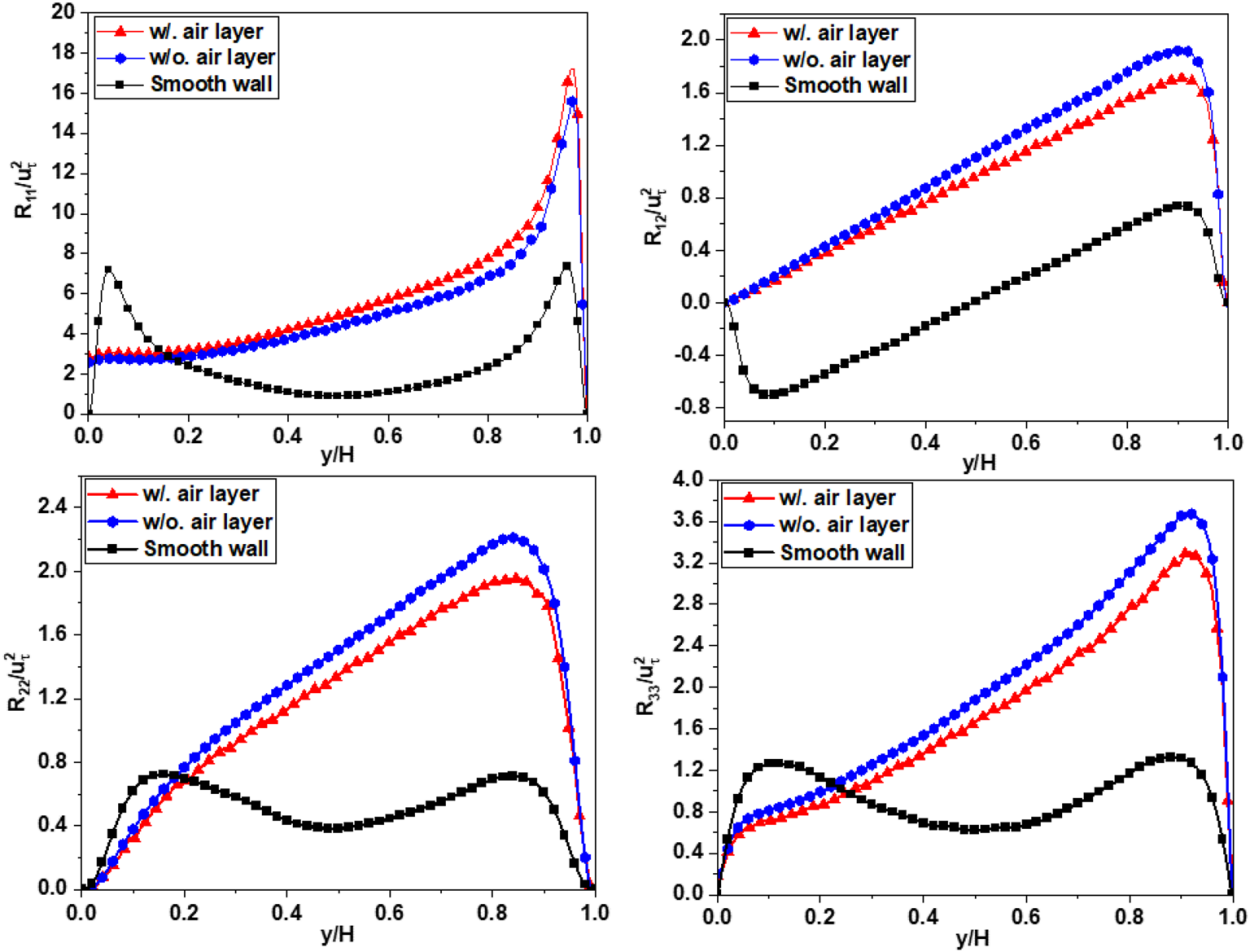
The Reynolds stress profile for Case 3.

Figures 16, 17, and 18 shows the planar averaged Reynolds stresses across all cases at a Reynolds number of *Re*_*τ*_ = 180, and all the Reynolds stresses are normalized by the square of the friction velocity $u_{\tau }^{2}$. The findings presented in these figures shed light on the prominent role of mean shear in influencing turbulence levels. The presence of a superhydrophobic surface is found to introduce a reduction in shear, which in turn leads to diminished turbulent production and lower overall turbulence levels across all measured shear stresses. The observed decrease in turbulence can be directly attributed to the reduced shear experienced at the superhydrophobic interface. The slip properties of this surface substantially mitigate the magnitude of shear, resulting in a significant drop in turbulence. The magnitude of this turbulence reduction is found to be closely associated with the extent of shear reduction induced by the slip effect on the superhydrophobic surface. Figures 16, 17 and 18 provide further insights into the influence of the air layer on the magnitude of the Reynolds stress at the interface. The presence of the air layer, resulting from the superhydrophobic surface, plays a significant role in modifying the Reynolds stress characteristics. Furthermore, the thickness of the air layer is identified as a crucial factor that impacts the magnitude of this stress alteration. As the thickness of the air layer varies, it directly influences the change in the Reynolds stress magnitude at the interface. The relationship between the thickness of the air layer and the corresponding change in Reynolds stress highlights the importance of considering the air layer thickness as a parameter in understanding and manipulating the turbulence dynamics in superhydrophobic systems. To calculate a prime-squared mean of the velocity field gives the Reynolds stress field for a statistically steady turbulent flow, typically refers to fluctuating component of velocity, usually denoted $u_{i}^{\left( k \right)}$ with i = 1, 2 and 3 for the *x*, *y*, and *z* components respectively, in the Reynolds decomposition of flow variables. These are obtained by subtracting the time-averaged velocity field from the instantaneous velocity field. The proccesing will be illustrated by equation ^23^:

15 $$\frac{1}{N}\mathop \sum \limits_{k = 1}^{N} \left( {U_{i}^{\left( k \right)} - \overline{{U_{i} }} } \right)^{2} = \frac{1}{N}\mathop \sum \limits_{k = 1}^{N} u_{i}^{{\left( k \right)^{2} }} = \left[ {\begin{array}{*{20}c} {\overline{{u_{1} u_{1} }} } & {\overline{{u_{1} u_{2} }} } & {\overline{{u_{1} u_{3} }} } \\ {\overline{{u_{2} u_{1} }} } & {\overline{{u_{2} u_{2} }} } & {\overline{{u_{2} u_{3} }} } \\ {\overline{{u_{3} u_{1} }} } & {\overline{{u_{3} u_{2} }} } & {\overline{{u_{3} u_{3} }} } \\ \end{array} } \right] = \left[ {\begin{array}{*{20}c} {R_{11} } & {R_{12} } & {R_{13} } \\ {R_{21} } & {R_{22} } & {R_{23} } \\ {R_{31} } & {R_{32} } & {R_{33} } \\ \end{array} } \right]$$

where $U_{i}^{\left( k \right)}$ represents the instantaneous velocity field *U*_*i*_ (*x*, *k*Δ*t*)), which is further decomposed into the time-averaged mean $\overline{{U_{i} }}$ and fluctuating components $u_{i}^{\left( k \right)}$,

16 $$U_{i}^{\left( k \right)} = \overline{{U_{i} }} + u_{i}^{\left( k \right)}$$

The diagonal elements of the Reynolds stress tensor represent the variances of the velocity components, while the off-diagonal elements represent the covariances.

Figure 16 shows the profile of the averaged Reynolds stresses in Case 1. The decrease in the values of *R*_11_, *R*_22_, *R*_33_, and *R*_12_ is observed near the bottom superhydrophobic surface (SHS), when compared to the case with a smooth channel. In contrast, these values increase at the wall surface with no-slip boundary condition (upper wall) for the simulation when ignoring the air layer. However, when the air layer is considered in the simulation, this trend persists for the parameters *R*_11_ and *R*_12_, while the parameters *R*_22_ and *R*_33_ experience a slight decrease near the upper wall. Similarly, this tendency is also evident in Cases 2 and 3, as depicted in Figs. 17 and 18, respectively. Furthermore, the extent of this difference observed is directly proportional to the level of drag reduction discussed earlier. These results illustrate that when it comes to controlling drag reduction, *R*_11_ and *R*_12_ exhibit a more pronounced impact compare to *R*_22_ and *R*_33_. This underscores the importance of taking into account *R*_11_ and *R*_12_ when optimizing the design and application of superhydrophobic surface for effective drag reduction strategies. Although the trend and difference are not large between the two cases, it helps us get closer to modeling the flow through the SHS surface, which is currently still a major barrier. In Case 3, which is different from other two cases, the Reynolds stress profiles show typical behavior similar with those of open channel flow with a free surface^41^. The Reynolds stress near the interface are damped to small or zero values without the peak and then are increased toward the opposite solid wall. The peak values near the solid wall are approximately three times larger than those for the smooth wall case.

### Budget of the turbulence kinetic energy

The transport equations for the Reynolds stresses are obtained by taking the ensemble average of the Navier–Stokes equations. This process involves deriving equations for the fluctuating stresses, and subsequently ensemble averaging them. In the case of incompressible turbulent flow, the nondimensionalized transport equations with $u_{\tau }^{4} /\nu$ with respect to the wall-shear velocity *u*_*τ*_ and the kinematic viscosity *ν* can be expressed as follows^41,42^:

17 $$\frac{{\overline{D}}}{Dt}\overline{{u_{i}^{\prime } u_{j}^{\prime } }} = P_{ij} + T_{ij} + \Pi_{ij} + D_{ij} - \epsilon_{ij}$$

where $\frac{{\overline{D}}}{Dt} = \frac{\partial }{\partial t} + U_{k} \frac{\partial }{{\partial x_{k} }}$, and the terms on the right-hand side of the above equation can be identified as follow:

18 $$P_{ij} = - \left[ {\overline{{u_{i}^{{\prime }} u_{j}^{{\prime }} }} U_{j,k} + \overline{{u_{j}^{{\prime }} u_{k}^{{\prime }} }} U_{i,k} } \right]\quad {\text{Production}}\,{\text{rate}}$$

19 $$\epsilon_{ij} = 2\overline{{u_{i,k}^{{\prime }} u_{j,k}^{{\prime }} }} \quad {\text{Dissipation}}\,{\text{rate}}$$

20 $$T_{ij} = - \left( {\overline{{u_{i}^{{\prime }} u_{j }^{{\prime }} u_{k}^{{\prime }} }} } \right),_{k} \quad {\text{Turbulent}}\,{\text{transport}}\,{\text{rate}}$$

21 $$D_{ij} = \left( {\overline{{u_{i}^{{\prime }} u_{j}^{{\prime }} }} } \right),_{kk} \quad {\text{Viscous}}\,{\text{diffusion}}\,{\text{rate}}$$

22 $$\Pi_{ij} = - \left( {\overline{{u_{i}^{{\prime }} p_{,j}^{{\prime }} + \overline{{u_{i}^{{\prime }} p_{,i}^{{\prime }} }} }} } \right)\quad {\text{Velocity}}\,{\text{pressure - gradient}}\,{\text{term}}$$

Repeated indices imply summation over the values 1, 2, and 3, while the indices (1, 2, 3) are used to represent the streamwise direction denoted as *x*^+^, the normal direction to the wall denoted as *y*^+^, and the spanwise direction denoted as *z*^+^, respectively. In the above equation, and throughout the subsequent discussion, p' represents a non-dimensional kinematic pressure. In a fully developed channel, the flow exhibits homogeneity in the streamwise direction (denoted as *x*^+^) and the spanwise direction (denoted as *z*^+^). The significant non-zero stresses in this scenario include $\overline{{u_{1}^{\prime } u_{1}^{\prime } }} ,\overline{{u_{2}^{\prime } u_{2}^{\prime } }} , \overline{{u_{3}^{\prime } u_{3}^{\prime } }}$, and $\overline{{u_{1}^{\prime } u_{2}^{\prime } }}$.

**Figure 19:**
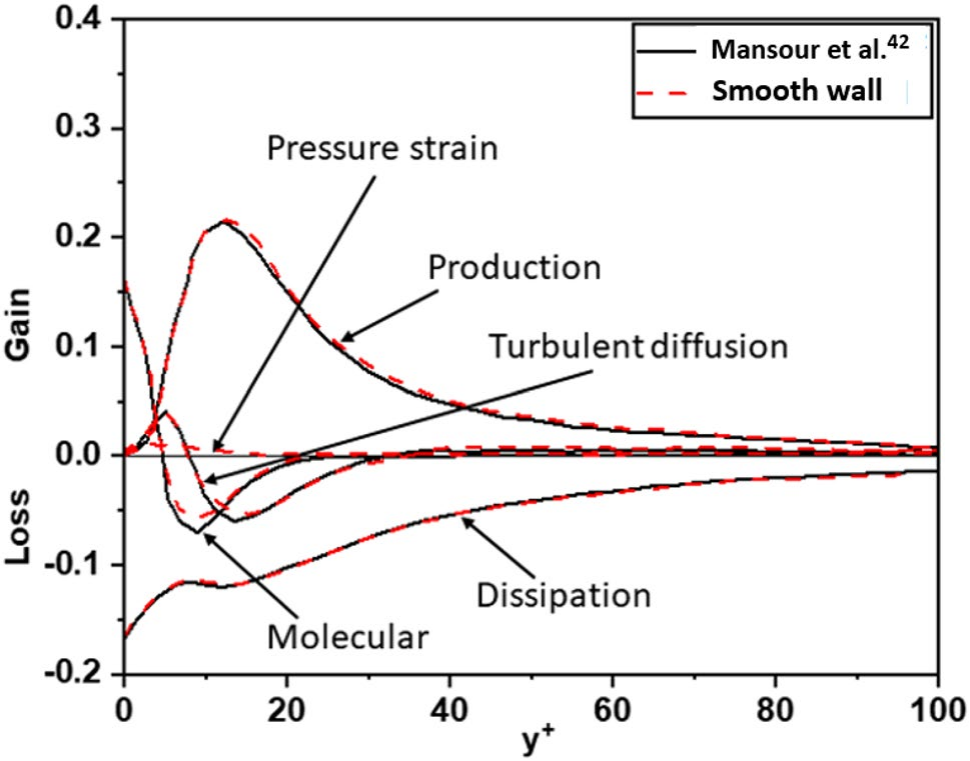
The comparison of terms in budget of turbulent kinetic for turbulent channel flow.

Figure 19 plots the budgets for the turbulence kinetic energy $k = \frac{1}{2} \left( { \overline{{u_{1}^{\prime } u_{1}^{\prime } }} + \overline{{u_{2}^{\prime } u_{2}^{\prime } }} + \overline{{u_{3}^{\prime } u_{3}^{\prime } }} } \right)$ such as production, dissipation, turbulent transport rate, turbulent diffusion, and pressure strain rate. The profiles for the different terms in these budgets (scaled by $u_{\tau }^{4} /\nu$) shows good agreement with the DNS (*Re*_*τ*_ = 180) results by Mansour et al.^42^.

**Figure 20:**
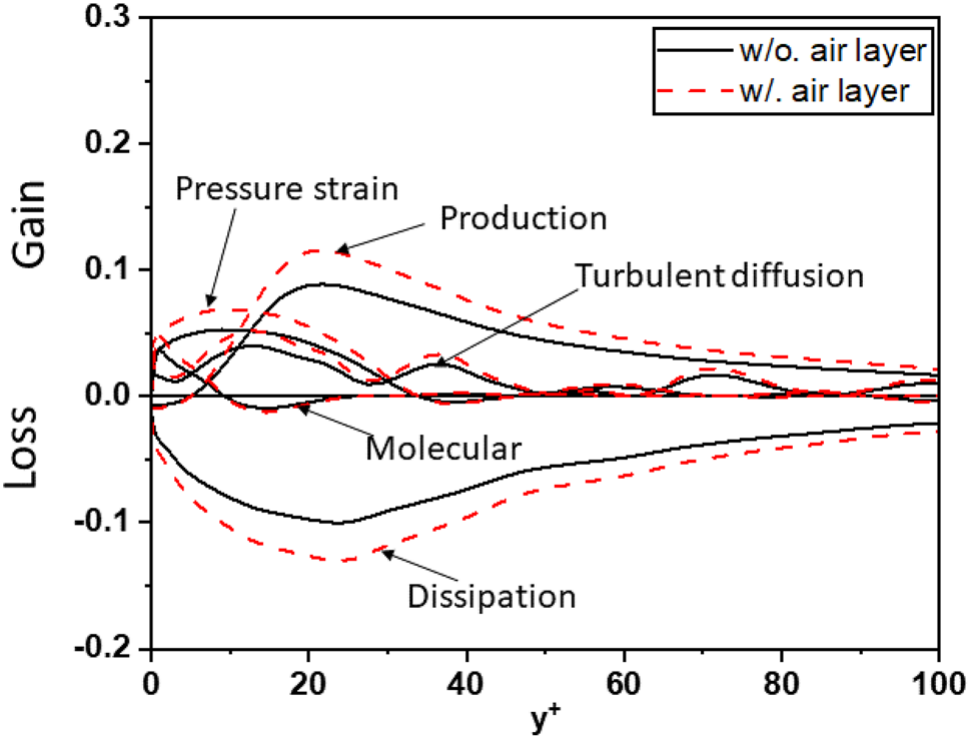
Budget of turbulent kinetic energy for Case 1.

**Figure 21:**
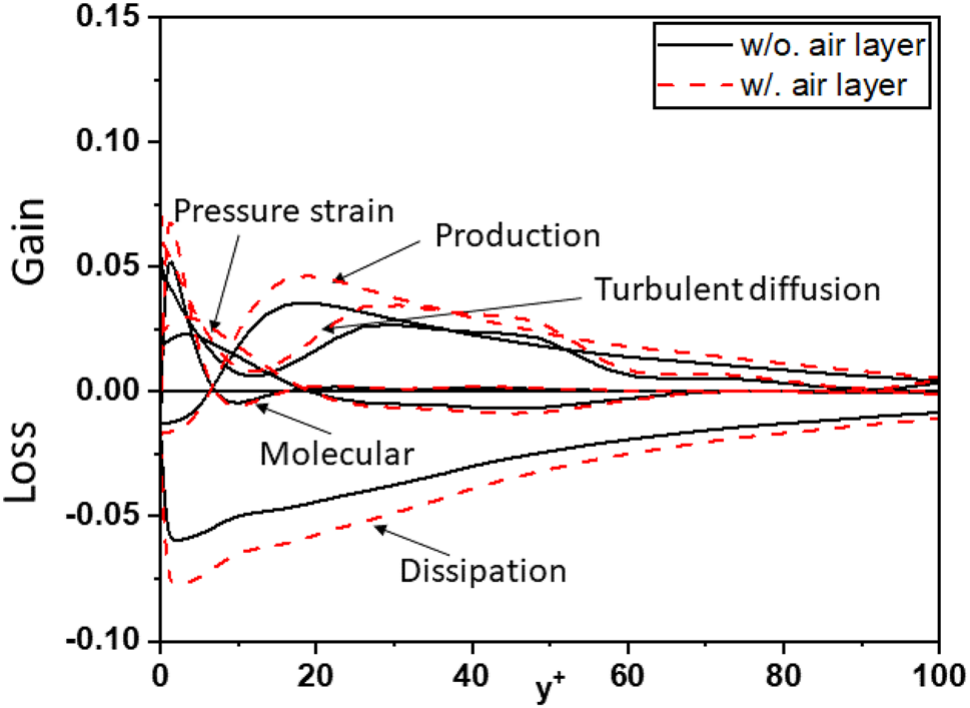
Budget of turbulent kinetic energy for Case 2.

**Figure 22:**
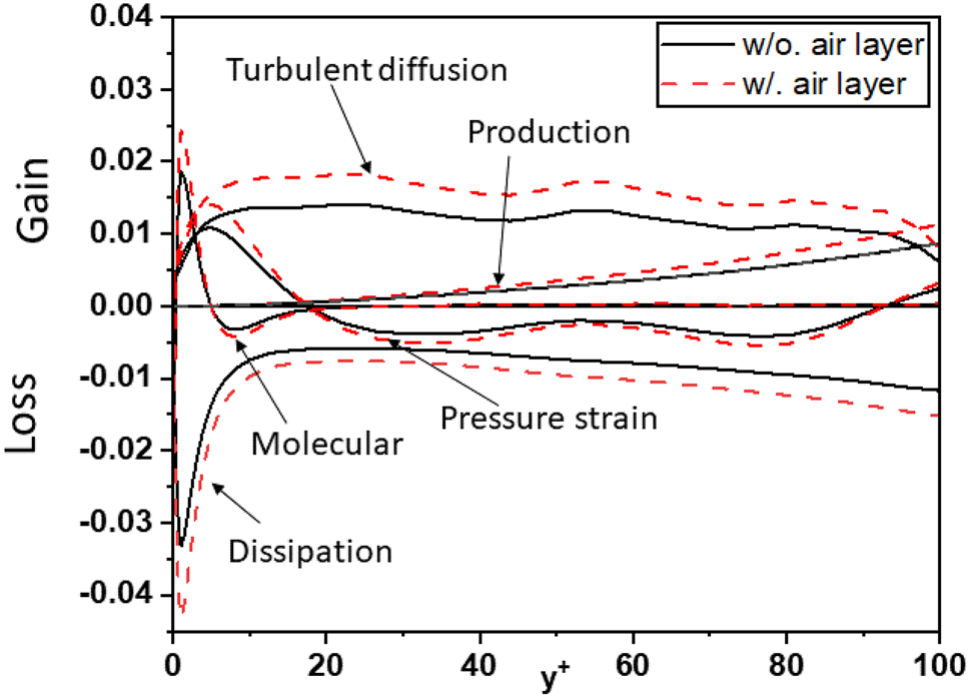
Budget of turbulent kinetic energy for Case 3.

To further strengthen the evidence regarding the influence of the air layer on turbulent kinetic energy (TKE), this study conducted a comprehensive analysis of the crucial components in the budget of turbulent kinetic energy for the investigated cases. Figures 20, 21, and 22 depict the components of the budget of turbulent kinetic energy for cases 1, 2, and 3, respectively. These figures provide valuable insights into the behavior of the components as the air layer thickness varies. Notably, the magnitudes of the components gradually decrease with increasing thickness of the air layer. This trend is consistent across all surveyed cases, regardless of whether the air layer is considered or not. Moreover, when examining the turbulent kinetic energy budget, certain components demonstrate distinct behavior. As the air layer thickness increases, the values of molecular diffusion and dissipation near the wall experience a significant increase, and approach the superhydrophobic surface (SHS). Additionally, pressure strain and turbulent diffusion contribute significantly to the overall process. This finding stands in contrast to the simulations conducted for the channel with a smooth wall, where these components do not hold the same level of significance in Fig. 19.

**Figure 23:**
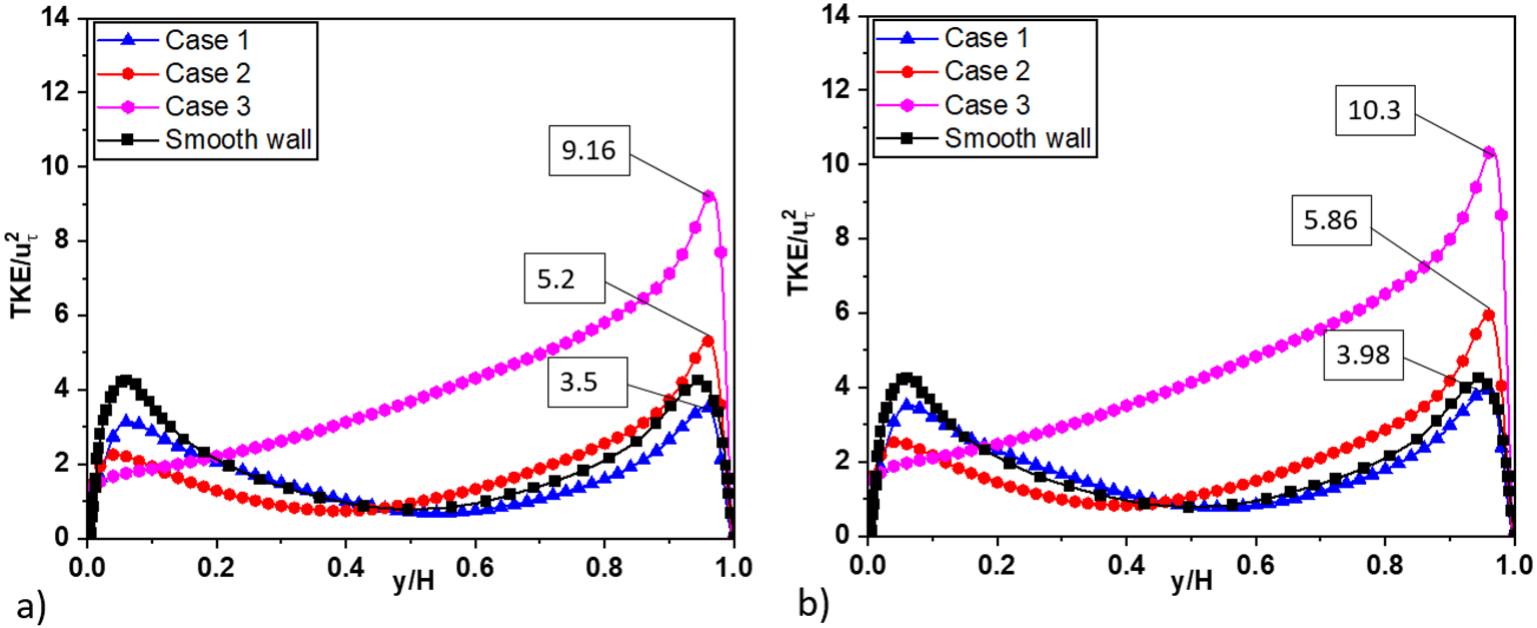
Profile of turbulent kinetic energy: (**a**) without air layer, (**b**) with air layer.

Figure 23a,b present compelling evidence regarding the impact of the air layer on turbulent kinetic energy. These figures clearly demonstrate that the presence of an air layer has a pronounced effect on TKE distribution. Specifically, the maximum TKE value near the superhydrophobic surface (SHS) and the smooth wall (upper wall) increases significantly when compared to the case where the air layer is ignored, with an approximate enhancement of 10–12%. Moreover, as the air layer thickness varies, the location of the maximum TKE value near the SHS shifts. As the air layer thickness increases, the maximum TKE value near the SHS decreases. Conversely, near the smooth wall (top wall), the maximum TKE value exhibits an opposite trend, and with thicker air layers, increases. These findings highlight the dynamic behavior of TKE distribution when SHS is employed, along with the influence of the air layer thickness. The observed shift in the location of the TKE peak towards the lower wall in the presence of SHS and thicker air layers further emphasizes the role of superhydrophobic surfaces in modifying turbulence characteristics and redistributing energy within the flow.

### Coherent structures near the wall using Q criterion

**Figure 24:**
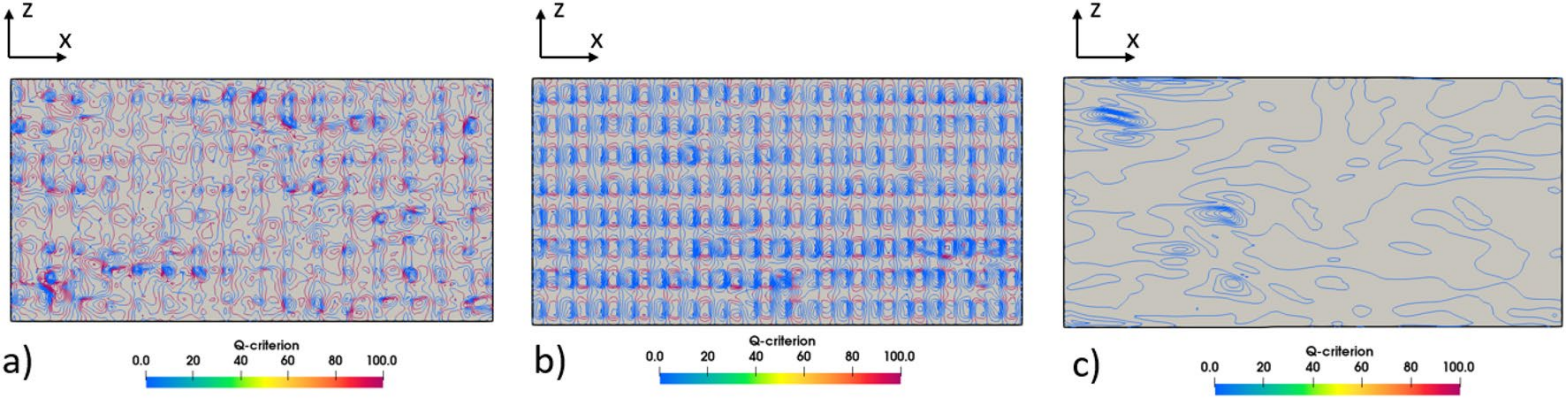
The Q contours at the interface for all investigated cases: (**a**) Case 1, (**b**) Case 2, (**c**) Case 3.

Due to the consistent tendency observed in the previous sections between the simulation cases considering the presence or absence of an air layer, the analysis in this section solely focuses on the results for the cases with an air layer. Although there are differences in the magnitudes of the values, the overall trend remains the same. The previous sections have provided substantial insights into perturbation structures through the analysis of DNS data for channels with superhydrophobic surfaces (SHS). Building upon this accumulated knowledge, this section specifically concentrates on analyzing the effect of the Q criterion on the coherent structures near the wall, such as vortices and streaks, in the presence of SHS. This analysis provides further clarity on the mechanisms driving flow behavior, aiding in the development of effective strategies for flow control and the optimization of superhydrophobic systems. For this issue, the Q criterion is used, Q being defined as reference^41^:

23 $$Q \equiv \frac{1}{2}\left( {u_{i,i}^{2} - u_{i,j} u_{j,i} } \right) = - \frac{1}{2}\left( {u_{i,j} u_{j,i} } \right) = \frac{1}{2}\left( {\Omega^{2} - {\text{S}}^{2} } \right)$$

where S and Ω are the symmetric and antisymmetric components of $\nabla u$, so Q represents the local balance between shear strain rate and vorticity magnitude. Figure 24 provides a visual representation of the Q contour at the interface for various air layer thicknesses. These figures collectively demonstrate that an increase in slip velocity both weakens the Q contours near the wall, and strongly influences the concentration, as determined by the shear rate.

**Figure 25:**
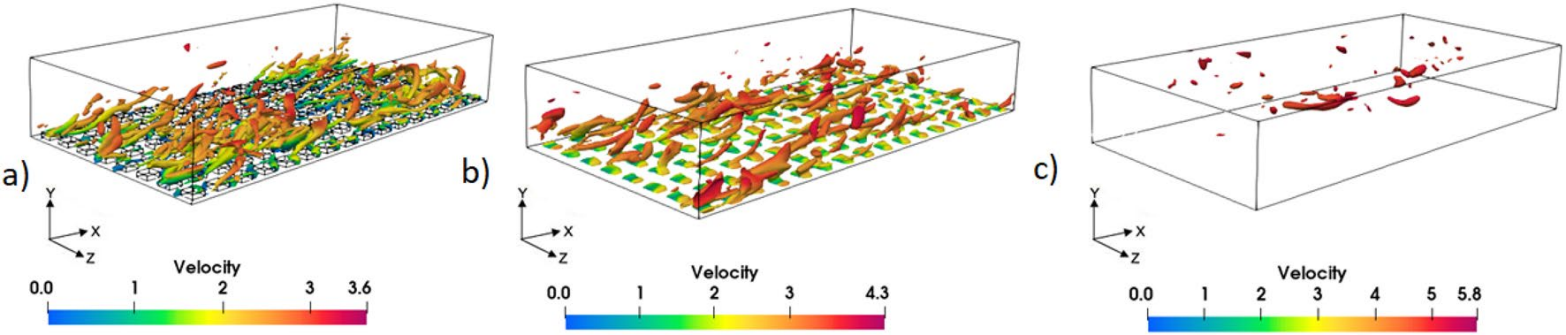
Contour of the coherent structure on the half-height channel with superhydrophobic surface at *Q* = 2 × 10^7^: (**a**) Case 1, (**b**) Case 2, and (**c**) Case 3.

Furthermore, Fig. 25 illustrates that higher slip velocities result in notable changes in the shape of these contours. Specifically, there is a decrease in streamwise stretching, and a reduction in the density of coherent structures near the superhydrophobic surface (SHS) at the same Q value. It is important to note that alterations in the length and direction of coherent structures can have a significant impact on turbulent oscillations. When the slip boundary condition leads to lower fluctuations, this signifies a reduction in turbulence production. As a result, the near-wall coherent structures are weakened, which is evident from the Q contour figures. These findings highlight the intricate relationship between slip velocity, Q contours, and the behavior of coherent structures near the SHS. By modifying slip conditions, researchers can strategically manipulate the density and characteristics of these coherent structures, thereby influencing turbulence levels and flow dynamics.

## Conclusion

This study investigated the influence of an air layer on drag reduction and turbulence dynamics in the channel flow with superhydrophobic surface by employing a fluid–fluid coupling method for water and air layer solvers. Simultaneously, the study takes into account variation in the air layer thickness and compares the results between simulations with and without the air layer. By making this comparison, the study offers insights into the overall effectiveness and precision of drag reduction levels in channels featuring superhydrophobic surfaces, along with an examination of how air layer thickness influences drag reduction. To be specific, the results of new approaching method can be summarized as follows:

- The study confirmed the effectiveness of the fluid–fluid coupling method in maintaining velocity profile integrity across the water–air interface. Also, a new approaching technique for simulating flow over superhydrophobic surface was introduced.
- The study analyzed the significant impact of the air layer on the velocity profile, resulting in lower velocities at the interface surface compared to simulations without the air layer. The crucial role of air layer thickness was identified in determining interface velocity with increasing thickness leading to higher velocities. The gradual increase in drag reduction with increasing air layer thickness was observed and the simulation models incorporating an air layer show smaller drag reduction compared to simulations without an air layer.
- The study also elucidated a direct correlation between air layer thickness and the alteration of Reynolds stress. The significance of Reynolds stress components *R*_11_ and *R*_12_ were emphasized in controlling drag reduction and optimizing superhydrophobic surface designs compared to other component of Reynolds stress. In addition, the study also analyzed the changes in turbulence kinetic energy components in both scenarios. It is revealed that the simulation incorporating an air layer leads to a change of about 10–12% of the turbulence kinetic energy compared to the simulation without the air layer. The density and length of the coherent structures decrease significantly as the thickness of the air layer increases and the slip velocity increases. This could be a signal for future SHS surface designs.

The results of the study will contribute to a better understanding of the mechanism of flow drag reduction across a superhydrophobic surface and move closer to modeling this physical phenomenon. In the future, it is possible to solve the above problem considering the impact of deformation of the interface surface.

## Data Availability

The data sets generated and thereafter analyzed would be available from the corresponding author upon reasonable request. The data are not publicly available due to necessity of special post-processing software to read raw data.
